# Uncovering spatiotemporal patterns of atrophy in progressive supranuclear palsy using unsupervised machine learning

**DOI:** 10.1093/braincomms/fcad048

**Published:** 2023-03-02

**Authors:** William J Scotton, Cameron Shand, Emily Todd, Martina Bocchetta, David M Cash, Lawren VandeVrede, Hilary Heuer, PROSPECT Consortium, 4RTNI Consortium, Alyssa A Costantini, Henry Houlden, Christopher Kobylecki, Michele T M Hu, Nigel Leigh, Bradley F Boeve, Brad C Dickerson, Carmela M Tartaglia, Irene Litvan, Murray Grossman, Alex Pantelyat, Edward D Huey, David J Irwin, Anne Fagan, Suzanne L Baker, Arthur W Toga, Alexandra L Young, Neil Oxtoby, Daniel C Alexander, James B Rowe, Huw R Morris, Adam L Boxer, Jonathan D Rohrer, Peter A Wijeratne

**Affiliations:** Dementia Research Centre, Department of Neurodegenerative Disease, UCL Queen Square Institute of Neurology, University College London, London WC1N 3AR, UK; Centre for Medical Image Computing, Department of Computer Science, University College London, London WC1V 6LJ, UK; Dementia Research Centre, Department of Neurodegenerative Disease, UCL Queen Square Institute of Neurology, University College London, London WC1N 3AR, UK; Dementia Research Centre, Department of Neurodegenerative Disease, UCL Queen Square Institute of Neurology, University College London, London WC1N 3AR, UK; Dementia Research Centre, Department of Neurodegenerative Disease, UCL Queen Square Institute of Neurology, University College London, London WC1N 3AR, UK; Department of Neurology, Memory and Aging Center, University of California, San Francisco, CA 94158, USA; Department of Neurology, Memory and Aging Center, University of California, San Francisco, CA 94158, USA; Department of Neuroimaging, Institute of Psychiatry, Psychology and Neuroscience, King’s College London, London SE5 8AF, UK; Centre for Medical Image Computing, Department of Computer Science, University College London, London WC1V 6LJ, UK; Centre for Medical Image Computing, Department of Computer Science, University College London, London WC1V 6LJ, UK; Cambridge University Department of Clinical Neurosciences and Cambridge University Hospitals NHS Trust, Medical Research Council Cognition and Brain Sciences Unit, Cambridge CB2 0QQ, UK; Department of Clinical and Movement Neurosciences, University College London Queen Square Institute of Neurology, London WC1N 3BG, UK; Movement Disorders Centre, University College London Queen Square Institute of Neurology, London WC1N 3BG, UK; Department of Neurology, Memory and Aging Center, University of California, San Francisco, CA 94158, USA; Dementia Research Centre, Department of Neurodegenerative Disease, UCL Queen Square Institute of Neurology, University College London, London WC1N 3AR, UK; Centre for Medical Image Computing, Department of Computer Science, University College London, London WC1V 6LJ, UK

**Keywords:** Subtype and Stage Inference, disease progression, progressive supranuclear palsy, biomarkers, machine learning

## Abstract

To better understand the pathological and phenotypic heterogeneity of progressive supranuclear palsy and the links between the two, we applied a novel unsupervised machine learning algorithm (Subtype and Stage Inference) to the largest MRI data set to date of people with clinically diagnosed progressive supranuclear palsy (including progressive supranuclear palsy–Richardson and variant progressive supranuclear palsy syndromes).

Our cohort is comprised of 426 progressive supranuclear palsy cases, of which 367 had at least one follow-up scan, and 290 controls. Of the progressive supranuclear palsy cases, 357 were clinically diagnosed with progressive supranuclear palsy–Richardson, 52 with a progressive supranuclear palsy–cortical variant (progressive supranuclear palsy–frontal, progressive supranuclear palsy–speech/language, or progressive supranuclear palsy–corticobasal), and 17 with a progressive supranuclear palsy–subcortical variant (progressive supranuclear palsy–parkinsonism or progressive supranuclear palsy–progressive gait freezing). Subtype and Stage Inference was applied to volumetric MRI features extracted from baseline structural (T1-weighted) MRI scans and then used to subtype and stage follow-up scans. The subtypes and stages at follow-up were used to validate the longitudinal consistency of subtype and stage assignments. We further compared the clinical phenotypes of each subtype to gain insight into the relationship between progressive supranuclear palsy pathology, atrophy patterns, and clinical presentation.

The data supported two subtypes, each with a distinct progression of atrophy: a ‘subcortical’ subtype, in which early atrophy was most prominent in the brainstem, ventral diencephalon, superior cerebellar peduncles, and the dentate nucleus, and a ‘cortical’ subtype, in which there was early atrophy in the frontal lobes and the insula alongside brainstem atrophy. There was a strong association between clinical diagnosis and the Subtype and Stage Inference subtype with 82% of progressive supranuclear palsy–subcortical cases and 81% of progressive supranuclear palsy–Richardson cases assigned to the subcortical subtype and 82% of progressive supranuclear palsy–cortical cases assigned to the cortical subtype. The increasing stage was associated with worsening clinical scores, whilst the ‘subcortical’ subtype was associated with worse clinical severity scores compared to the ‘cortical subtype’ (progressive supranuclear palsy rating scale and Unified Parkinson’s Disease Rating Scale). Validation experiments showed that subtype assignment was longitudinally stable (95% of scans were assigned to the same subtype at follow-up) and individual staging was longitudinally consistent with 90% remaining at the same stage or progressing to a later stage at follow-up.

In summary, we applied Subtype and Stage Inference to structural MRI data and empirically identified two distinct subtypes of spatiotemporal atrophy in progressive supranuclear palsy. These image-based subtypes were differentially enriched for progressive supranuclear palsy clinical syndromes and showed different clinical characteristics. Being able to accurately subtype and stage progressive supranuclear palsy patients at baseline has important implications for screening patients on entry to clinical trials, as well as tracking disease progression.

## Teaser

Scotton *et al*. apply Subtype and Stage Inference, a machine learning algorithm, to a cross-sectional MRI data to identify two distinct and stable spatiotemporal subtypes of atrophy progression in progressive supranuclear palsy (PSP). These subtypes provide unique insights into disease progression in PSP.

## Graphical Abstract

**Graphical Abstract:**
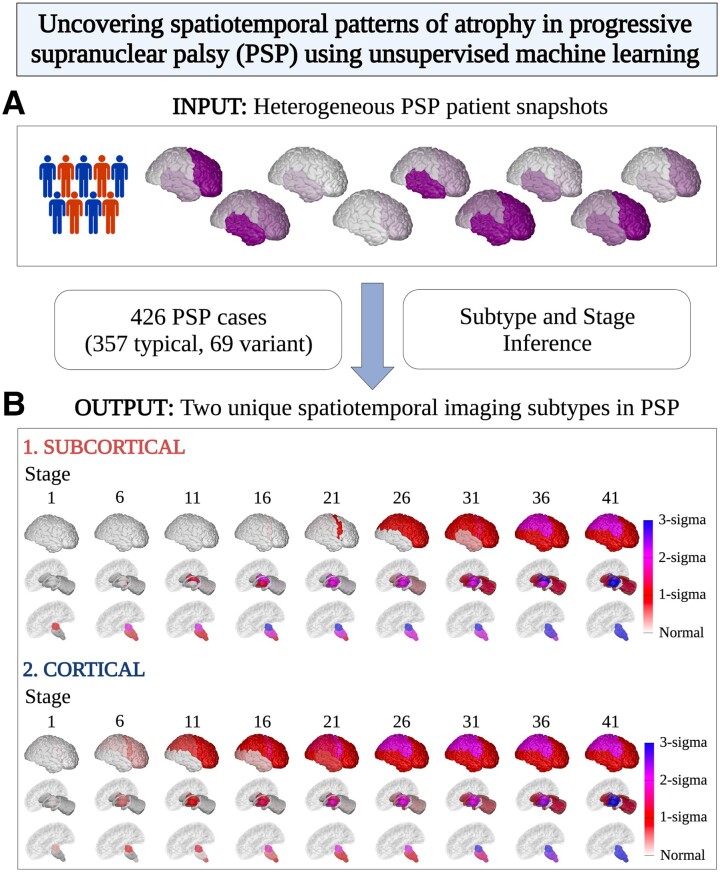

## Introduction

Many neurodegenerative diseases are complicated by poor clinico-pathological correlation, with the underlying pathology often manifesting as a range of different, and often overlapping, clinical syndromes. Defining disease phenotypes based on common underlying biological mechanisms, as opposed to the clinical phenotype, is an important step towards enriching clinical trials with patients that are most likely to benefit from the medicine being investigated, especially as therapeutics increasingly target these biological mechanisms. Recent advances in machine learning have enabled analysis of multidimensional data to classify and stage groups with similar data-driven features (such as spatiotemporal atrophy patterns on MRI^1^) rather than just on common clinical features, providing new tools to tackle the problem of clinical heterogeneity.

Progressive supranuclear palsy (PSP), a neurodegenerative disease defined pathologically by the aggregation and spread of 4-repeat tau protein in neurons, astrocytes, and oligodendrocytes,^2^ shows significant differences in severity and neuroanatomical distribution of pathology,^3^ resulting in a range of clinical phenotypes involving language, behaviour, and movement abnormalities.^4^ No effective disease-modifying treatment has yet been proven for PSP, despite increasingly available clinical trials.^5,6^ Clinical progression appears to be dependent on progressive spreading of the four-repeat (4R) tau pathology within the brain, with a recent pathology staging system^3^ defining six sequential stages of progression for the most common clinical phenotype PSP–Richardson syndrome (PSP-RS), starting in the pallido–nigro–luysian system and spreading rostrally via the striatum and amygdala to the cerebral cortex (frontal > temporo-parietal > occipital) and caudally to the medulla oblongata, pons, and cerebellum.^7^ Although the molecular pathogenic basis for clinical variation is still poorly understood, this study suggests that differences in tau burden and different tau cytopathologies may distinguish clinical subtypes.

The Movement Disorder Society 2017 PSP diagnostic criteria^8^ were introduced to try and account for variant PSP clinical phenotypes (vPSP) and increase the sensitivity and specificity for diagnosis of early PSP pathology. The criteria categorize symptomatology into four clinical domains (ocular motor dysfunction, postural instability, akinesia, and cognitive dysfunction), with differing combinations of these symptoms defining a range of clinical PSP syndromes. Although the most common clinical presentation of PSP is Richardson syndrome (PSP-RS), vPSP may account for up to 50% of individuals with PSP pathology.^4,9,10^ The vPSP syndromes include subcortical variants [including PSP–parkinsonism (PSP-P) and PSP–primary gait freezing (PSP-PGF)] and cortical variants [PSP–frontal (PSP-F), PSP-corticobasal syndrome (PSP-CBS), and PSP–speech/language syndrome (PSP-SL)]. These new criteria have higher sensitivity than the previous National Institute of Neurological Disorders and Stroke and Society for Progressive Supranuclear Palsy criteria (87.9% versus 45.5%),^11,12^ and the ‘suggestive of PSP’ clinical category significantly increases the sensitivity for early identification of patients with PSP pathology.^13^ One potential issue with the Movement Disorder Society diagnostic criteria as first defined was that patients can be assigned multiple phenotypes according to clinical symptomatology.^14^ The introduction of the multiple allocation extinction rules^13^ helps to allocate patients to one phenotype, though its application to clinically diagnosed PSP patients appears to lead to an over representation of PSP-RS versus vPSP syndromes such as PSP-P and PSP-PGF.^15-17^ This has important implications for clinical trials given that the subcortical variants (PSP-P and PSP-PGF) have better survival with longer disease durations (PSP-P 9 years and PSP-PGF 13 years versus PSP-RS 6–7 years) and slower rates of disease progression.^9,17-23^ Improved quantification of the progression of pathological brain changes across the PSP phenotypic spectrum in living patients will be essential to the success of future therapeutic trials.^7,24^

A probabilistic data-driven modelling approach (event-based modelling) has been used to characterise the in-vivo sequence of brain atrophy in PSP-RS,^25^ where the order of regional atrophy broadly mirrors the sequential spread of tau pathology proposed by Kovacs *et al*.^3^ However, the event-based modelling assumes sample homogeneity, making it unsuitable to investigate the full spectrum of PSP phenotype heterogeneity. Subtype and Stage Inference (SuStaIn), an unsupervised machine learning algorithm,^1^ has been developed to identify data-driven disease subtypes with distinct temporal progression patterns and can do so using only cross-sectional data. The trained model can then be used to subtype and stage new individuals. This ability to disentangle both ‘phenotypic’ and ‘temporal’ heterogeneity from cross-sectional biomarkers distinguishes SuStaIn from traditional approaches that focus on either one or the other. The SuStaIn subtypes account for temporal heterogeneity, enabling more accurate subtype assignment than traditional clustering algorithms. This algorithm has been successfully applied to Alzheimer’s disease,^1,26^ multiple sclerosis,^27^ and genetic frontotemporal dementia,^1,28^ providing important insights into distinct data-driven subtypes of disease progression. This type of disease progression modelling approach is ideally suited to disentangling the clinical and pathological heterogeneity of PSP.

In this study, we applied the SuStaIn algorithm to cross-sectional MRI data from a large international cohort of clinically diagnosed PSP patients (including PSP-RS and vPSP syndromes), to identify imaging subtypes with distinct sequences of atrophy progression. We validated the observed subtypes and stages using a subset of longitudinal imaging data. Finally, we characterized the clinical features of each subtype to gain insight into the relationship between PSP pathology, atrophy patterns, and clinical presentation.

In this paper, we use ‘syndrome’ when referring to the PSP clinical syndrome/phenotype (as defined in the Movement Disorder Society 2017 diagnostic criteria^8^) and ‘subtype’ when referring to MRI-based subtypes identified by SuStaIn.

## Materials and methods

### Participants and clinical data collected

Clinical and MRI data from individuals with a clinical diagnosis of ‘possible’ or ‘probable’ PSP (including PSP-RS and vPSP syndromes) as per the Movement Disorder Society 2017 PSP diagnostic criteria^8^ were collected from seven main sources; the 4R Tauopathy Imaging Initiative Cycle 1 (4RTNI1; ClinicalTrials.gov: NCT01804452),^29,30^ the 4R Tauopathy Imaging Initiative Cycle 2 (4RTNI2; ClinicalTrials.gov: NCT02966145), the davunetide randomized control trial (ClinicalTrials.gov: NCT01056965),^31^ the salsalate clinical trial (ClinicalTrials.gov: NCT02422485),^32^ the young plasma clinical trial (ClinicalTrials.gov: NCT02460731),^32^ the PROgressive Supranuclear Palsy CorTico-Basal Syndrome Multiple System Atrophy Longitudinal Study (PROSPECT; ClinicalTrials.gov: NCT02778607),^33^ and the University College London Dementia Research Centre FTD cohort. Controls for *z*-scoring imaging data were collected from three sources with available cross-sectional volumetric MRI: the Frontotemporal Lobar Degeneration Neuroimaging Initiative data set (FTLDNI; http://4rtni-ftldni.ini.usc.edu/), PROSPECT, and the University College London Dementia Research Centre FTD cohort. Controls were defined as no known diagnosis of a neurological or neurodegenerative condition and no known history of memory complaints. Further details on the 4RTNI2 cohort are included in the Supplementary material, and details of demographics and clinical information by cohort are provided in Supplementary Table 1. Information pertaining to recruitment, diagnostic criteria, and MRI scanner acquisition protocols for the other cohorts has been provided previously.^25^ Appropriate ethics was applied for and approved via each of the individual trial and research ethics committees. To be included in this study, all participants needed to have, as a minimum, a clinical diagnosis of PSP (PSP-RS or vPSP), a baseline T_1_ volumetric MRI on a 1.5 or 3 tesla scanner, and basic demographic data (gender and age at time of scan). Clinical rating scale scores [PSP rating scale, Unified Parkinson Disease Rating Scale (UPDRS), Schwab and England Activities of Daily Living scale (SEADL), and Montreal Cognitive Assessment (MoCA) or Mini-mental State Examination (MMSE) at baseline and follow-up], pathology at autopsy, and follow-up scans were also included if available. As detailed in a previous work,^25^ original trial analyses failed to show any treatment effect (including no change in volumetric MRI measurements) in the salsalate, young plasma,^32^ and davunetide^31^ trials, so data were combined from each study’s treatment and placebo arms. Longitudinal data were used to validate the consistency of SuStaIn’s subtype and stage assignments at follow-up.

Given that the PROSPECT and 4RTNI2 trials only assessed cognitive function using the MOCA (as opposed to the MMSE for the other trials), raw MOCA scores were converted to MMSE scores using the validated method used by Lawton *et al*.^34^ For missing data in clinical scales, an adjusted mean score was used if at least 80% of the assessment was complete.^33^

### MRI acquisition and image processing

The acquisition and postprocessing procedures have been described previously.^25^ Briefly, cortical and subcortical structures were parcellated using the geodesic information flow algorithm (GIF),^35^ which automatically extracts regions based on the Neuromorphometrics atlas (Neuromorphometrics Inc.), using an atlas propagation and label fusion strategy.^36,37^ Subregions of the cerebellum were extracted using GIF based on the Diedrichsen atlas.^38^ The medulla, pons, superior cerebellar peduncles (SCP), and midbrain were subsequently segmented using a customised version of a module available in FreeSurfer to accept the GIF parcellation of the whole brainstem as input.^39^ Volumes for 24 grey matter regions were calculated; four brainstem (medulla, pons, SCP, and midbrain), three cerebellar (cerebellar cortex, dentate nucleus, and vermis), nine subcortical (thalamus, globus pallidus, caudate, putamen, ventral diencephalon, thalamus, hippocampus, amygdala, and nucleus accumbens), and eight cortical (basal forebrain, cingulate, frontal anterior, frontal posterior, insula, temporal, parietal, and occipital) regions. Regions that had a right and left label were combined. A list of the GIF subregions included in each cortical region is included in Supplementary Table 2. The total intracranial volume was calculated using SPM12 v6225 (Statistical Parametric Mapping, Wellcome Trust Centre for Neuroimaging, London, UK) running under MATLAB R2012b (Math Works, Natick, MA, USA) (Scotton 2022; 40). All segmentations were visually inspected to ensure accurate segmentation.

All volumes were corrected for total intracranial volume, scanner field strength (1.5 T or 3 T), scanner manufacturer, age at baseline scan, and sex, using linear regression on the control population and then propagating this model to the PSP population, to generate covariate-adjusted regional volumes. To confirm that age effects on regional brain volumes had been successfully regressed out, linear models were fit to assess for any residual association between individual covariate-adjusted regional volumes and age at scan.

### Biomarker selection and data preparation

A biomarker in this study was defined as an image-based regional volume extracted using GIF. To reduce dimensionality, we carried out pairwise comparisons between healthy volunteers and patients at baseline visit and selected MRI regions whose differences between groups were associated with a moderate to large effect size (Cohen’s *d* effect size of ≥0.6 for standardized mean differences between the cases and controls). Applying this threshold to the regional volumes segmented by GIF (see ‘MRI acquisition and image processing’) resulted in selection of 20 regions of interest (ROI) that were then included for downstream analysis (Supplementary Table 3). Adjusted regional volumes for the ROI were converted into *z*-scores relative to the control group by subtracting the mean of the control group from each patient’s ROI volume and dividing by the standard deviation of the control group. Given that regional brain volumes decrease with disease progression, the *z*-scores become negative as the disease progresses; *z*-scores were therefore multiplied by −1, to give positive *z*-scores that increase with disease progression. The *z*-scored data was then used as input to SuStaIn.

### Subtype and Stage Inference

SuStaIn is a probabilistic machine learning algorithm that simultaneously clusters individuals into groups (subtypes) and infers a trajectory of change associated with each group; that trajectory defines the disease stage (degree of disease progression within a subtype) of each individual within the corresponding group. SuStaIn requires only cross-sectional data as input, although it can exploit longitudinal data for training if available. The details of the algorithm have been published previously^1^ and applied to a range of different diseases.^26-28,40^ Each subtypes’ progression pattern is described using a piecewise linear *z*-score model, expressing a trajectory with a series of stages that each correspond to a single biomarker (regional brain volume in this case) reaching a new *z*-score. Importantly, the number of SuStaIn stages is determined by the number of biomarkers (the product of the number of ROIs and number of *z*-score thresholds per ROI) provided as input. SuStaIn optimizes both the subtype membership and the ordering in which different biomarkers reach different *z*-scores in each subtype (for example one, two or three standard deviations away from the control mean for that ROI) using a data likelihood function.

Only clinically diagnosed PSP cases were used to fit SuStaIn. Supplementary Table 4 provides a summary of the *z*-score settings, Markov Chain Monte Carlo iterations and number of random starting sequences used for the SuStaIn algorithm. Model uncertainty was estimated using 100 000 Markov Chain Monte Carlo iterations, and in the single-cluster expectation maximization procedure, the single-cluster sequence was optimized from 24 different random starting sequences to find the maximum likelihood solution. The optimal number of subtypes was determined using information criteria calculated through ten-fold cross-validation (cross-validation information criteria), to balance internal model accuracy with model complexity.^1^ Where there was no strong evidence for an additional subtype in the model using the cross-validation information criteria, we assessed the average log-likelihood across folds for the additional subtype and, if there was no improvement, selected the most parsimonious model, i.e. the model with fewer subtypes.

Finally, the fitted SuStaIn model was used to calculate the probability that each individual falls at each stage of each subtype, and individuals were assigned to their maximum likelihood stage and subtype based on their baseline scan (as described in Young *et al*.^1^). The subtype progression patterns identified by SuStaIn were visualized using BrainPainter software,^41^ modified to include the brainstem segmentations.

### Assigning individuals to subtypes and stages

Individuals’ stage was computed based on their average stage, weighted by the probability of belonging to each stage of each subtype. Individuals that were assigned to either SuStaIn stage 0 (i.e. no atrophy on imaging compared to controls) or stage 41 (end stage, i.e. all ROI maximum atrophy) were labelled ‘no subtype’. All other individuals were labelled as ‘subtypable’ and were then assigned to their most probable subtype.

### Statistical analyses

For all analyses, PSP cases were stratified into PSP-RS, PSP–subcortical, and PSP–cortical groups based on their baseline clinical diagnosis. The PSP–subcortical group includes individuals with PSP-P and PSP-PGF; the PSP–cortical group includes cases with PSP-SL, PSP-F, and PSP-CBS.

#### Clinical phenotype and baseline characteristics

Pairwise comparisons of baseline characteristics were performed between all PSP cases and controls, PSP syndrome (PSP-RS, PSP–cortical, and PSP–subcortical) versus all PSP cases, and each PSP syndrome against each other, using -tests for continuous variables and $\chi^{2}$ tests for categorical variables. Statistical significance was reported at a level of *P* < 0.05 and at the Bonferroni corrected level of *P* < 0.001 to correct for multiple comparisons (44 items).

#### Association between subtype assignment and covaria*t*tes

To assess for any residual association between covariates (total intracranial volume, scanner field strength, scanner manufacturer, age at baseline scan, sex, cohort, and SuStaIn stage) and the SuStaIn subtype, a logistic regression model was fit to the data using the ‘lm()’ function from the ‘stats’ package (version 3.6.2).

#### Subtype characterisation

Overall differences between subtypes were first assessed independently of stage, with individuals classified as ‘no subtype’ (i.e. stage 0 or 41) excluded from analysis. To compare whether there were any differences between subtypes, we performed *t*-tests for continuous variables and $\chi^{2}$ tests for categorical variables (and *post hoc* pairwise comparisons for clinical syndrome versus the SuStaIn subtype using the ‘chisq.multicomp()’ function from the ‘RVAideMemoire’ R package version 0.9-81-2).

To test for associations between clinical scores (PSP rating scale, UPDRS, SEADL, and MMSE) and subtype, we accounted for the SuStaIn stage, age, and sex by fitting a linear model (clinical test score∼subtype + stage + age + sex) for each clinical test score. Statistical significance was reported uncorrected at a level of *P* < 0.05 and at the Bonferroni corrected level of *P* < 0.005 for demographic variables (11 items) and for clinical scores (10 items), to account for multiple comparisons.

To assess the average stage by clinical syndrome by the SuStaIn subtype, a one-way ANOVA was performed (mean stage∼PSP syndrome + SuStaIn baseline subtype) with the ‘aov()’ function the ‘stats’ package (version 3.6.2). Tukey *post hoc* significant differences were than calculated to identify the level of significance.

All statistical analyses were performed either in R (version 4.0.5) or Python (version 3.7.6).

### Longitudinal validation

The SuStaIn model fitted on the cross-sectional baseline data was used to assign the maximum likelihood stage and subtype to all follow-up scans (at all time points). These scans were used to validate the stability of subtypes and to assess stage progression, based on the hypothesis that individuals should remain in the same subtype but should advance to higher stages over time (or at least remain at the same stage). Subtype stability was defined as the proportion of individuals assigned to the same subtype at follow-up(s) or progressed from stage 0 into a subtype, compared to the total number of individuals. Stage progression was assessed by comparing the SuStaIn stage at baseline and follow-up(s) for all individuals. Specifically, we calculated the proportion of individuals that advanced or stayed at the same stage at follow-up scan.

## Results

### Participants

Table 1 summarizes the key baseline clinical features for individuals included in the study. For a breakdown of this data by each contributing cohort, please refer to Supplementary Table 1. We collected a large imaging cohort of PSP cases; 1083 MRI images were included (after quality control) from a total of 716 individuals: 426 with a clinical diagnosis of PSP (with 367 follow-up scans) and 290 controls. Of the PSP cases, 357 (84%) were diagnosed with PSP-RS, 52 (12%) with a PSP–cortical syndrome (PSP-SL, PSP-F, or PSP-CBS), and 17 (4%) with a PSP–subcortical syndrome (PSP-P or PSP-PGF). After coming to post-mortem, 31 (7%) of the PSP cases had a pathological diagnosis, of which 29 (94%) showed 4R tau pathology consistent with PSP, whereas two cases that presented with PSP-RS had non-PSP tau pathology (one corticobasal degeneration and one globular glial tauopathy). Given that the focus of this study was understanding the clinical heterogeneity of clinically diagnosed PSP, both cases were included in the analysis.

**Table 1:** Clinical phenotypes and baseline characteristics

	Study group									
		PSP by subgroup								
				PSP–cortical				PSP–subcortical		
	Controls	All	PSP-RS	PSP-SL	PSP-F	PSP-CBS	All cortical	PSP-P	PSP-PGF	All subcortical
Baseline, *n* (fu visits)	290	426 (367)	357 (329)	35 (23)	10 (2)	7 (0)	52 (25)	7 (8)	10 (5)	17 (13)
Sex, % female	57%	49%	49%	46%	40%	71%	48%	14%	60%	41%
Age at first scan, y	62.5 (9.4)	68.5 (6.8)^d^	68.1 (6.6)	68.6 (7.7)	68.8 (8.6)	77.6 (2.7)	69.9 (7.9)	71.5 (7.7)	71.6 (7.1)	71.5 (7.1)
Age at first symptom, y^a^		64.1 (7.5)	63.5 (7.2)	64.1 (8.1)	64.6 (7.7)	72.7 (3.8)	65.4 (8.0)	64.5 (10.3)	66.6 (5.4)	65.7 (7.6)
Disease duration, y^a,b^		4.7 (3.2)	4.6 (3.3)	4.5 (2.8)	4.19 (3.0)	4.9 (2.0)	4.5 (2.7)	6.9 (3.5)	5.1 (2.9)	5.8 (3.2)
Pathology, % PSP		31 (94%)^c^	26 (92%)	-	1 (100%)	1 (100%)	2 (100%)	4 (100%)	1 (100%)	5 (100%)
PSP rating scale score		34.9 (15.0)	37.2 (13.2)^e,h,i^	11.9 (10.8)	19.5 (13.2)	56.4 (11.9)	20.6 (20.0)^f,h^	33.8 (7.89)	23 (10.4)	26.3 (10.7)^e,i^
History		7.8 (3.9)	8.3 (3.6)^h,i^	3.4 (3.3)	5 (2.8)	10.7 (3.4)	4.8 (4.2)^f,h^	8.5 (3.9)	4.4 (3.1)	5.7 (3.7)^i^
Mentation		3.4 (2.6)	3.5 (2.3)^i^	1.8 (1.7)	5.2 (4.2)	4.8 (2.6)	2.8 (2.2)	4 (2.6)	1.0 (0.9)	1.9 (1.3)^e,i^
Bulbar		2.5 (1.8)	2.7 (1.8)^h^	1.8 (1.7)	1.2 (1.4)	2.3 (1.9)	1.8 (1.7)^e,h^	2.7 (2.5)	1.6 (1.1)	1.9 (1.7)
Ocular motor		7.6 (4.0)	8.4 (3.5)^e,h,j,l^	1.3 (0.6)	2.4 (1.9)	10.4 (2.4)	2.9 (2.2)^f,h,j,l^	5.8 (3.3)	4.6 (3.5)	4.9 (3.4)^e,h,j,l^
Limb motor		4.3 (2.9)	4.5 (2.7)^i^	2.4 (2.2)	1.2 (1.0)	10.9 (2.9)	3.5 (3.2)	3.5 (1.3)	2.9 (1.4)	3.1 (1.3)^e,i^
Gait and midline		9.2 (5.2)	9.9 (4.7)^h^	2.2 (2.1)	3.6 (3.2)	17.3 (3.0)	4.8 (3.5)^f,h,k^	9.5 (2.1)	8.6 (3.5)	8.9 (3.1)^k^
SEADL		54.8 (24.8)	53.8 (24.0)^i^	66.2 (33.1)	51.4 (13.5)	26.6 (13.5)	58.5 (30.2)	57.5 (20.6)	68.9 (14.5)	65.4 (16.6)^e,i^
UPDRS		27.3 (18.0)	29.3 (14.7)^g^	9.3 (8.9)	11.5 (10.9)	65.6 (12.2)	18.8 (17.6)^g,k^	35.5 (9.3)	36.4 (14.7)	36.2 (12.9)^e,k^
MMSE		25.5 (3.8)	25.7 (3.7)^j^	27 (2.6)	21.9 (5.3)	19.4 (4.0)	24.7 (4.6)^l^	22 (0.8)	22.6 (1.3)	22.4 (1.2)^f,j,l^

Values are mean (SD), apart from gender, % female; baseline *n* (*n* follow-up visits); and pathology *n* (% PSP). Pairwise comparisons between groups were performed using *t*-tests for continuous variables and $\chi^{2}$ tests for categorical variables.

CBD, cortico-basal degeneration; GGT, globular glial tauopathy; MMSE, Mini-Mental State Examination; PSP-C, PSP–cortical (includes PSP–frontal, PSP–speech/language disorder, and PSP–corticobasal syndrome); PSP-RS, PSP–Richardson syndrome; PSP-SC, PSP–subcortical (includes PSP–parkinsonism and PSP–progressive gait freezing); SEADL, Schwab and England Activities of Daily Living; UPDRS, Unified Parkinson’s Disease Rating Scale.

a Note incomplete data for disease duration/age at first symptom.

b Time from first symptom to first scan.

c Two cases of not PSP pathology (1 CBD, 1 GGT).

d PSP all versus controls. Statistically significant at *P* < 0.05, corrected for multiple comparisons (44 comparisons, *P* < 0.00114).

e PSP (subgroup) versus PSP–all. Statistically significant at *P* < 0.05, uncorrected for multiple comparisons.

f PSP (subgroup) versus PSP–all. Statistically significant at *P* < 0.05, corrected for multiple comparisons (44 comparisons, *P* < 0.00114).

g PSP-RS versus PSP-C. Statistically significant at *P* < 0.05, uncorrected for multiple comparisons.

h PSP-RS versus PSP-C. Statistically significant at *P* < 0.05, corrected for multiple comparisons (44 comparisons, *P* < 0.00114).

i PSP-RS versus PSP-SC. Statistically significant at *P* < 0.05, uncorrected for multiple comparisons.

j PSP-RS versus PSP-SC. Statistically significant at *P* < 0.05, corrected for multiple comparisons (44 comparisons, *P* < 0.00114).

k PSP-C versus PSP-SC. Statistically significant at *P* < 0.05, uncorrected for multiple comparisons.

l PSP-C versus PSP-SC. Statistically significant at *P* < 0.05, corrected for multiple comparisons (44 comparisons, *P* < 0.00114).

Overall, the PSP cases at baseline had an older average age compared to controls {68.5 years [standard deviation (SD) ± 6.8] versus 62.5 years (SD 62.5), *P* < 0.001}, though they were matched for gender. We confirmed that despite the difference in age between cases and controls, age effects had been effectively regressed out of the regional covariate-adjusted volumes for both groups: cases (Supplementary Fig. 1 and Supplementary Table 5) and controls (Supplementary Fig. 2 and Supplementary Table 6). There were significant differences in baseline clinical scores between the different clinical PSP phenotypes. The highest PSP rating scale score (measure of motor predominant disease burden) was seen in PSP-RS [37.2 (SD ± 13.2)], followed by PSP subcortical syndromes [26.3 (SD ± 10.7)], with PSP cortical syndromes being the least impaired [20.6 (SD ± 20)]. There was, however, a large variation in this score for the cortical syndromes [PSP-SL 11.9 (SD ± 10.8), PSP-F 19.5 (SD ± 13.2), and PSP-CBS 56.4 (SD ± 11.9)]. In keeping with the increased motor predominant disease burden (higher PSP rating scale score) in the PSP-RS and PSP–subcortical cases, the UPDRS was significantly higher in these cases versus PSP–cortical cases [PSP–subcortical 36.2 (SD ± 12.9), PSP-RS 29.3 (SD ± 14), PSP–cortical 18.8 (SD ± 7.6), *P* < 0.05 for each comparison]. The PSP–subcortical cases had a better MMSE score on average compared to the PSP-RS and PSP–cortical syndromes [22.4 (SD ± 1.2) versus 25.7 (SD ± 3.7) and 24.7 (SD ± 4.6), *P* < 0.001 for each comparison]. There was no significant difference in MMSE between the latter two syndromes.

### Spatiotemporal subtypes of PSP

SuStaIn was fit using PSP cases only, based on the rationale that PSP is a rare disease, and it is very unlikely for our cohort of controls to have asymptomatic PSP. Indeed, it is more likely that the controls would have a more common neurodegenerative disorder such as Alzheimer’s disease rather than PSP, and we did not want this to confound the Subtype and Stage Inference estimation hence the exclusion.

**Figure 1:**
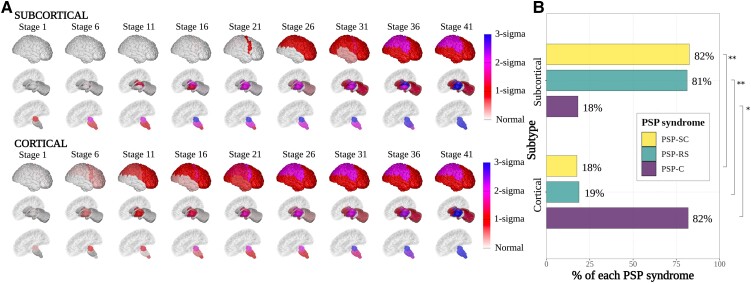
**Subtype progression patterns of PSP atrophy identified by Subtype and Stage Inference (SuStaIn). (A)** Spatial distribution and severity of atrophy at each SuStaIn stage by subtype. Each row (subcortical top, cortical bottom) represents a subtype progression pattern identified by SuStaIn consisting of a set of stages at which brain volumes in PSP cases reach different *z*-scores relative to controls. **(B)** Assignment of PSP clinical syndromes to each SuStaIn subtype. Size of bar (*x*-axis) represents percentage of cases labelled with that PSP syndrome assigned to that SuStaIn subtype (*y*-axis). A Pearson chi-square test was performed with *post hoc* pairwise comparisons for clinical syndrome versus the SuStaIn subtype using the chisq.multicomp() function from the RVAideMemoire R package version 0.9-81-2 [$\chi^{2}$ (2, *N* = 406) = 81.8, *P* = 2.2 × 10^−16^]. *Statistically significant at *P* < 0.05, uncorrected for multiple comparisons. **Statistically significant at *P* < 0.05, corrected for multiple comparisons. Visualizations in **A** were generated using the BrainPainter software,^41^ modified to include the brainstem segmentations.

SuStaIn identified two imaging subtypes with distinct patterns of regional atrophy evolution (Fig. 1A and Supplementary Fig. 3 for the positional variance diagrams). Supplementary Fig. 4 shows the log-likelihoods after 10-fold cross validation with the associated cross-validation information criteria demonstrating that the two-subtype model was the most parsimonious. Based on the earliest MRI abnormalities seen in the SuStaIn-defined trajectories, we labelled the first the ‘subcortical’ subtype and the second the ‘cortical’ subtype. The ‘subcortical’ subtype (75% of the cases) has atrophy in the midbrain followed by the other brainstem structures (medulla, pons, and SCP) and the ventral diencephalon at early SuStaIn stages. The atrophy then progresses caudally to the dentate nucleus of the cerebellum and rostrally to the thalamus and lentiform nucleus (globus pallidus and putamen) before spreading to the cortex (after stage 13). Cortical atrophy progresses in an anterior to posterior direction, beginning in the insula and posterior frontal lobe, before spreading to the temporal, parietal, and finally the occipital lobe. The ‘cortical’ subtype (25% of cases) has more generalized atrophy in the early SuStaIn stages, with the midbrain and insula affected first and then the frontal lobes (posterior > anterior), thalamus, ventral diencephalon, and the basal ganglia all affected at a similar time (before stage 13). Interestingly, the end stage atrophy pattern is similar for both subtypes.

Overall, 20 of the 426 scans (5%) were not subtypable at baseline and so were excluded from subtype *post hoc* analyses. Three of these individuals had a clinical diagnosis of PSP-RS and were at stage 41 and 17 were at stage 0 [9 PSP-RS and 8 PSP–cortical (all PSP-SL)].

A logistic regression model was fit to assess for any residual association between the SuStaIn subtype, regressed covariates, and SuStaIn stage (SuStaIn subtype∼SuStaIn stage + TIV + age at first scan + sex + scanner + scanner field strength + scanner manufacturer). This demonstrated a remaining association between the SuStaIn subtype and age at first scan (*z* = 2.8, *P* = 6 × 10^−3^), General Electric 3T scanner (*z* = −3.0, *P* = 3 × 10^−3^), Phillips 3T scanner (*z* = −2.5, *P* = 0.01), and the 4RTNI2 cohort (*z* = 3.6, *P* = 3 × 10^−4^). There was no dependency of the subtype on stage (*z* = −0.1, *P* = 0.91) with a similar distribution of stages across each subtype (Supplementary Fig. 5A).

### Association between PSP clinical syndromes and subtype

We compared the subtype assignments (‘subcortical’ versus ‘cortical’) for clinical PSP syndromes (PSP-RS versus PSP–cortical and PSP–subcortical). Figure 1B and Table 2 show the percentage of each of these clinical syndromes assigned to each subtype. The ‘subcortical’ SuStaIn subtype was significantly enriched for PSP-RS and PSP–subcortical syndromes; 81% of PSP-RS cases (*P* = 2 × 10^−6^) and 82% of PSP–subcortical cases (*P* = 0.007) were assigned to the SuStaIn ‘subcortical’ subtype. The ‘cortical’ SuStaIn subtype was enriched for the PSP–cortical syndromes; 81% of PSP–cortical syndromes (*P* = 2 × 10^−5^) were assigned to the SuStaIn ‘cortical’ subtype.

**Table 2:** Comparison of demographics, clinical diagnosis, and test scores between subtypes

	Subcortical subtype	Cortical subtype	*P*-value
All scans, *n*	321 (75.4)	105 (24.6)	
Subtypable scans, *n*	302 (74.4)	104 (25.6)	0.07^a^
Average subtype probability^b^	0.94 (0.1)	0.85 (0.2)	<0.005^c^
Sex, % female	49%	48%	0.82
Age at first scan, y	68.1 (6.3)	70.0 (8.1)	0.02
Age at first symptom, y^d^	63.8 (6.9)	65.5 (8.3)	0.14
Disease duration, y^d,e^	4.4 (3.1)	5.0 (3.2)	0.26
PSP syndrome, *n*			<0.005^c^
PSP-RS	280 (81%)	65 (19%)	–
PSP-C	8 (18%)	36 (82%)	–
PSP-SC	14 (82%)	3 (18%)	<0.005^c^
PSP rating scale	37.0 (13.6)	30.3 (16.9)	<0.005^c^
SEADL	53.7 (23.8)	56.3 (26.6)	0.39
UPDRS	30.0 (16.2)	22.8 (21.0)	<0.005^c^
MMSE	25.5 (3.6)	24.9 (4.2)	0.20

Values are mean (SD) or *n* (%), apart from sex = % female. Pairwise comparisons between groups were performed using *t*tests for continuous variables and $\chi^{2}$ tests for categorical variables.

PSP-C, PSP–cortical (includes PSP–frontal, PSP–predominant speech/language disorder, and PSP–predominant corticobasal syndrome); PSP-RS, PSP–Richardson syndrome; PSP-SC, PSP–subcortical (includes PSP–parkinsonism and PSP–progressive gait freezing); SEADL, Schwab and England Activities of Daily Living; UPDRS, Unified Parkinson’s Disease Rating Scale; MMSE, Mini-Mental State Examination.

a All scans versus subtypable scans.

b Subtype probability = the probability of assignment for an individual case to the given subtype.

c Statistically significant at *P* < 0.05, corrected for multiple comparisons (11 comparisons, *P*-value < 0.005).

d Note incomplete data for disease duration/age at first symptom.

e Time from first symptom to first scan.

### Subtype demographics and clinical characteristics

Table 2 gives an overview of demographics, clinical diagnosis, and test scores by subtype. With an average subtype probability assignment of 0.94 compared to 0.85 for the ‘cortical’ subtype (*t* = −6.5, *P* < 0.005), 74% of the subtypable scans were assigned to the ‘subcortical’ subtype. Those in the ‘subcortical’ subtype were both slightly younger at symptom onset [63.8 (SD ± 6.9) years versus 65.5.0 (SD ± 8.3)] and at time of baseline scan [68.1 (SD ± 6.3) years versus 70.0 (SD ± 8.1)], though this did not reach statistical significance in either case. Pairwise comparisons of clinical scores demonstrated that PSP rating scale scores [37.0 (SD ± 13.6) versus 30.3 (SD ± 16.9), *t* = −3.7, *P* < 0.005] and UPDRS [30.0 (SD ± 16.2) versus 22.8 (SD ± 21.0)] were higher (i.e. more severe motor predominant disease burden) in the ‘subcortical’ subtype. Average MMSE was similar between subtypes [‘subcortical’ subtype 25.5 (SD ± 3.6) versus ‘cortical’ subtype 24.9 (SD ± 4.2), *t* = −1.3, *P* < 0.20].

**Figure 2:**
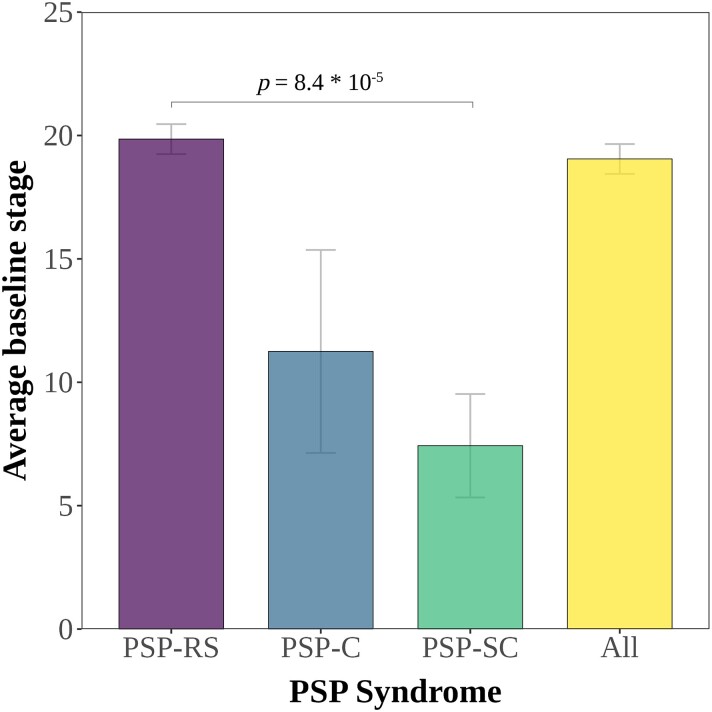
**Average stage of PSP clinical syndromes assigned to the ‘subcortical’ SuStaIn subtype.** Mean SuStaIn stage for PSP syndrome, with associated standard error bars. ANOVA: PSP syndrome = 12.1, *P* = 8 × 10^−6^; SuStaIn subtype = 1 × 10^−4^, *P* = 0.97). Tukey *post hoc* honest significance test: in ‘subcortical’ SuStaIn subtype PSP-RS versus PSP-SC estimate = −12.4, *P* = 8.4 × 10^−5^. No other *post hoc* comparisons significant.

The average stage for subtypable individuals within each subtype was similar [19.0 (SD ± 10.5) for ‘subcortical’ versus 18.3 (SD ± 9.1) for ‘cortical’, *β* = 8 × 10^−6^, *P* = 0.85] (Supplementary Table 7). However, PSP–subcortical cases (82%) assigned to the ‘subcortical’ SuStaIn subtype were on average at a lower stage [7.4 (SD ± 5.8)] compared to PSP-RS cases assigned to either the ‘subcortical’ [19.9 (SD ± 10.2), *P* < 0.003] (Fig. 2) or the ‘cortical’ subtype [18.9 (SD ± 8.7), *P* < 0.003]. We then tested whether the PSP–subcortical and PSP-RS cases assigned to the ‘subcortical’ subtype showed differences in the rate of progression (defined as change in subtype per year). PSP–subcortical cases in the ‘subcortical’ subtype progressed on average 0.66 stages per year, compared to 1.86 stages per year for the PSP-RS cases (*t* = 2.49, 95% CI 0.1–2.4, *P* = 0.046). One PSP–subcortical case progressed from stage 12 (no cortical involvement) to stage 14 (insula and posterior frontal lobe abnormal), whilst two cases had more extensive cortical involvement at baseline (stage 16 and stage 26, respectively, at baseline, and stage 16 and stage 27, respectively, at follow-up).

### Association between stage, subtype, and disease severity

Table 3 shows the relationship between clinical test scores and the SuStaIn subtype and stage across all subtypable cases, accounting for age at first scan and sex. The performance on the total PSP rating scale score (and history, bulbar, ocular motor, and gait midline subscores) was worse in the ‘Subcortical’ subtype and related to the stage, suggesting that these scores decline with disease progression in both subtypes, but the overall scores are worse in the ‘subcortical’ subtype. The UPDRS score was worse in the ‘subcortical’ subtype but unrelated to the SuStaIn stage, suggesting that test performance shows a stronger decline with disease progression in the ‘subcortical’ subtype. Worsening SEADL, PSP rating scale limb motor, and mentation subscores were associated with increasing SuStaIn stage, suggesting that these scores decline with disease progression in both subtypes. All these associations survived Bonferroni correction for multiple comparisons. MMSE showed no difference between subtypes and was not associated with the SuStaIn stage.

**Table 3:** Comparison of adjusted clinical scores between subtypes

	SuStaIn subtype		SuStaIn stage			
	*t*-value	*P*-value	*t*-value	*P*-value	Subtype with worse score	Change with SuStaIn stage
PSP rating scale score						
*Total*	−4.12	5 × 10^−5^b^^	5.21	3 × 10^−7^b^^	Subcortical subtype	Worsens
*History*	−3.98	8 × 10^−5^b^^	4.21	3 × 10^−5^b^^	Subcortical subtype	Worsens
*Mentation*	−0.86	0.39	3.74	2 × 10^−4^b^^		Worsens
*Bulbar*	−2.17	0.03^a^	3.42	7 × 10^−4^b^^	Subcortical subtype	Worsens
*Ocular motor*	−4.56	7 × 10^−6^b^^	3.90	1 × 10^−4^b^^	Subcortical subtype	Worsens
*Limb motor*	−0.40	0.69	3.25	0.001^b^		Worsens
*Gait and midline*	−4.02	7 × 10^−5^b^^	3.12	0.002^b^	Subcortical subtype	Worsens
SEADL	1.03	0.30	−5.57	5 × 10^−8^b^^		Worsens
UPDRS	−2.67	0.009^b^	1.70	0.08	Subcortical subtype	
MMSE	−1.03	0.31	−1.42	0.16		

Linear model of clinical score ∼ subtype + stage + age + sex.

SEADL, Schwab and England Activities of Daily Living; UPDRS, Unified Parkinson’s Disease Rating Scale; MMSE, Mini-Mental State Examination.

a Statistically significant at *P* < 0.05, uncorrected for multiple comparisons.

b Statistically significant at *P* < 0.05, corrected for multiple comparisons (10 items, *P* < 0.0125).

### Longitudinal consistency of subtypes

Given that we used cross-sectional MRI data to infer distinct longitudinal trajectories, evaluating how well longitudinal data fits the SuStaIn model is a key aspect of validation. We tested this in two ways: firstly, by assessing whether subtype assignments were longitudinally stable and, secondly, by testing whether individuals progressed to later stages at follow-up. A total of 355 follow-up scans (355/367) were subtypable at follow-up from 289 PSP cases (224 had one follow-up scan, 64 had two, and one individual had three). Mean follow-up time was 0.91 years with a SD of 0.38 years.

Overall, the SuStaIn subtype assignments showed good stability at follow-up (Table 4), with 95% (347 out of 367 visits) either remaining in the same subtype or progressing from the normal-appearing (not subtypable) group to the ‘subcortical’ or ‘cortical’ subtypes. Ninety-seven per cent (265/273) of PSP scans assigned to the ‘subcortical’ subtype at previous scan remained in that subtype at follow-up; 2% (five scans) switched to the ‘cortical’ subtype at follow-up scan, whilst 1% (three scans) reverted to ‘normal’ (not subtypable). Of those scans assigned to the ‘cortical’ subtype, 96% (78/81) showed stable subtype assignment, whilst 4% (three scans) switched to the ‘subcortical’ subtype.

**Table 4:** Longitudinal consistency of subtype assignments

	Classification at follow-up visit		
Classification at previous visit	Normal appearing^a^	Subcortical subtype	Cortical subtype
**Normal appearing^a^**	9 (69%)	**3 (23%)^b^**	**1 (8%)^b^**
**Subcortical subtype**	3 (1%)	**265 (97%)^b^**	5 (2%)
**Cortical subtype**	0 (0%)	3 (4%)	**78 (96%)^b^**

a Normal appearing = not subtypable. Note that this only includes 13 individuals that were not subtypable at baseline and had a follow-up scan.

b An observation is longitudinally consistent if individuals remain in the same group or progress from the normal-appearing group to the subcortical or cortical subtype at follow-up visit. Entries indicate the number of visits *n*, with the % of the total individuals in classification at previous visit in classification at follow-up in brackets. Longitudinally consistent observations highlighted in bold.

**Figure 3:**
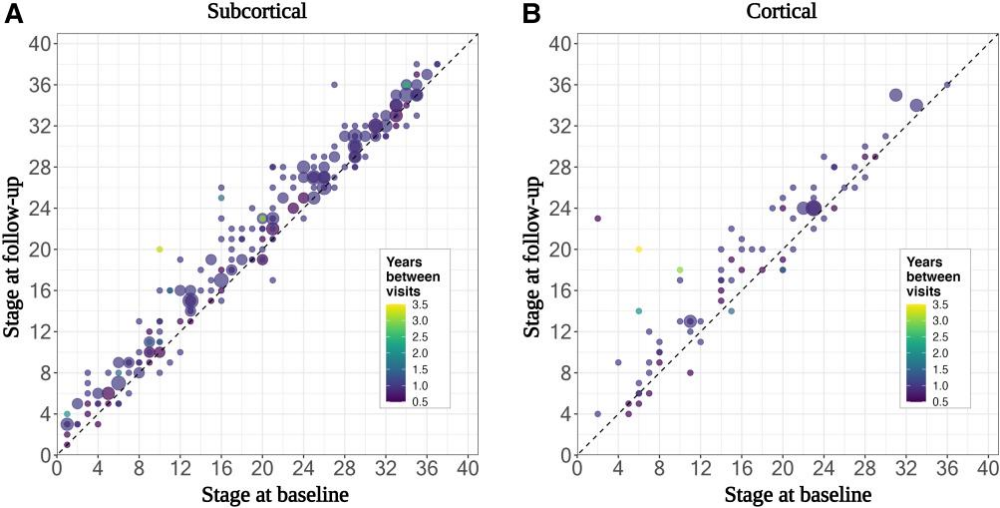
**Stage progression at follow-up visits by the SuStaIn subtype.** Scatter plots of **(A)** the ‘subcortical’ subtype and **(B)** the ‘cortical’ subtype showing the predicted stage at baseline (*x*-axis) versus the predicted stage at follow-up scan (*y*-axis) for those PSP cases with a follow-up scan (*n* = 355). The area of the circle is weighted by the number of scans at each point, and the colour of the circle represents the time (years) between visits.

We next tested how the SuStaIn stage progressed over time (Fig. 3) by comparing the assigned stage at follow-up to the baseline stage. As expected, the majority of individuals (90%) either progressed in stage (75%, 318/355) or stayed at the same stage (15%, 53/355), i.e. are on or above the line y=x. For those individuals assigned to the ‘subcortical’ subtype (Fig. 3A), 92% stayed at the same stage or progressed (17% and 75%, respectively); for those diagnosed with PSP-RS, 92% stayed at the same stage or progresses, for PSP–cortical 100%, and for PSP–subcortical 100%. In the ‘cortical’ subtype (Fig. 3B), 83% stayed at the same stage or progressed (9% and 74%, respectively) in the ‘cortical’ subtype; the breakdown for clinical phenotypes assigned to this subtype was 83.9% for PSP-RS, PSP–subcortical 100%, and PSP–cortical 77%.

## Discussion

The clinical heterogeneity of PSP is increasingly recognized^7,8^ and although post-mortem studies^3,9,10^ suggest that this heterogeneity is related to differences in the severity and neuroanatomical distribution of pathology, there is an urgent need to better delineate this variability in vivo. To this end, we applied SuStaIn to a large PSP MRI data set, encompassing the spectrum of PSP clinical syndromes, and empirically identified two subtypes characterized by distinct temporal patterns of atrophy. We referred to these two subtypes as ‘subcortical’ and ‘cortical’ based on the earliest regions to show abnormality. Clinical scores of disease severity worsened with increasing stage and the ‘subcortical’ subtype was associated with more severe disease compared to the ‘cortical’ subtype, as measured by the PSP rating scale. It is worth noting, however, that the PSP rating scale was originally designed to assess disease severity in PSP-RS and as such is heavily weighted towards measuring motor predominant disease burden. It is therefore unlikely to be a good measure of overall disease burden in the cortical PSP syndromes,^42^ and the scores in these cases relative to the PSP-RS/PSP–subcortical variants need to be interpreted with caution. As expected, the ‘cortical’ subtype was enriched for patients clinically diagnosed with PSP–cortical variants (PSP-CBS, PSP-F, and PSP-SL). The ‘subcortical’ subtype was enriched for patients clinically diagnosed with both PSP-RS and PSP–subcortical variants (PSP-P, PSP-PGF), though on average, the PSP–subcortical variants were at a lower subtype stage with a slower rate of progression compared to the PSP-RS cases. The distinct patterns of MRI atrophy in these subtypes provides unique insights into disease mechanisms across the disease course and supports the stratification of patients by subtype in clinical trials.

The ‘subcortical’ and ‘cortical’ SuStaIn subtypes share some common early features (Fig. 1A and Supplementary Fig. 3), though they are distinguished by much earlier cortical involvement in the latter. Both subtypes demonstrate early involvement of the midbrain and ventral diencephalon (which includes the subthalamic nucleus and substantia nigra), in keeping with previous post-mortem studies,^3,9^ and several neuropathological studies of incidental or early-stage PSP cases.^43-50^ In contrast to the ‘subcortical’ subtype, the ‘cortical’ subtype has concomitant atrophy in the insula, frontal lobes, thalamus, and basal ganglia in these early stages. The subsequent cortical atrophy in the ‘cortical’ subtype then progresses to the parietal, occipital, and cingulate and finally the temporal lobe. At equivalent stages, the subcortical atrophy progresses to the medulla, SCP, dentate, and globus pallidus, followed by the amygdala, and finally the caudate and cerebellar cortex. In contrast, in the ‘subcortical’ subtype, atrophy has already progressed through the whole brainstem, SCP, and dentate nucleus, before it reaches the insula and posterior frontal lobe (supplementary motor cortex). Once it reaches the cortex, the sequence of cortical atrophy is broadly similar to the ‘cortical’ subtype.

The sequence of atrophy in the ‘subcortical’ subtype broadly mirrors the sequential spread of tau pathology proposed by Kovacs *et al*. in their post-mortem PSP-RS staging system and is consistent with other *in vivo* disease progression models of PSP-RS.^25,51^ Whilst Scotton *et al*.^25^ used an event-based modelling approach on PSP-RS cases to identify this sequence, Saito *et al*.^51^ applied SuStaIn to a small cohort of PSP-RS and CBS cases. They found that the optimal model consisted of two subtypes, with one subtype associated with CBS and the other to PSP-RS. Given the small sample size of the Saito study and the absence of any vPSP cases in the sample, they were unable to extract finer grained information on PSP heterogeneity.^25,51^

An important difference in our study was that in addition to the majority of PSP-RS cases (81%) being assigned to the ‘subcortical’ subtype, the same was also true for the PSP–subcortical (PSP-P and PSP-PGF) cases (82%). The implication is that atrophy in PSP–subcortical variants progresses along the same trajectory as PSP-RS cases. Previous work shows that PSP-P and PSP-PGF cases develop similar clinical phenomenology to PSP-RS cases in the later stages of the disease course,^52-54^ albeit at a slower rate^17,54^ resulting in longer survival times.^20^  *Post hoc* analysis of the ‘subcortical’ SuStaIn subtype supports this, with the PSP-SC cases at an earlier stage in the sequence than PSP-RS cases (Fig. 2) (7.4 versus 19.9, *t* = 12.4, *P* = 8.4 × 10^−5^), with a slower stage progression per year (0.66 versus 1.86, *t* = 2.49, 95% CI 0.1–2.4, *P* = 0.046).

Traditional cross-sectional imaging studies show that the PSP–subcortical variants usually have less severe atrophy in the midbrain, medulla, and SCP relative to PSP-RS cases.^2,33,55-59^ Although this may seem at odds with SuStaIn assigning PSP-RS and PSP–subcortical to the same trajectory, it can be reconciled by the finding that at the average stage for PSP–subcortical variants (7.4), the *z*-score has only reached 2 sigma (two standard deviations from controls) for the midbrain and 1 sigma for the medulla and SCP, versus 3, 2, and 2 sigma, respectively, by stage 20 (the average stage for the PSP-RS cases). This suggests that on average, PSP-SC cases have less atrophy in those regions at the time of baseline scan compared to PSP-RS cases, which would be consistent with the cross-sectional imaging findings that do not account for disease stage heterogeneity. It is currently unclear as to why the PSP–subcortical variants show a less aggressive disease, though it may be at least partly due to protective genetic variants.^60^

There was also a strong assignment of PSP–cortical cases (PSP-F, PSP-SL, and PSP-CBS) to the ‘cortical’ subtype, which drove a lower average PSP rating scale and UPDRS score compared to the ‘subcortical’ subtype. As previously discussed, the ‘cortical’ subtype had early cortical atrophy predominantly affecting the insula and frontal lobes alongside the subcortical involvement, which is in keeping with cross-sectional MRI studies of PSP–cortical variants.^33,55,61,62^ Although there are few longitudinal imaging studies of these variants due to their rarity, a recent retrospective cohort of PSP-SL demonstrated that the majority of these cases developed symptoms typical of PSP-RS as their disease progressed.^62^ Our data supports this finding given that the end-stage atrophy pattern of the ‘cortical’ SuStaIn subtype is very similar to the ‘subcortical’ atrophy pattern. Further work is required to better understand what is driving the difference between the ‘subcortical’ and ‘cortical’ subtype atrophy patterns, especially early in the disease trajectory. The early cortical involvement in the ‘cortical’ subtype may, at least in part, be due to higher astroglial or oligodendroglial pathology relative to neuronal tau pathology early in the disease course,^3^ though this is an area of ongoing research.

## Limitations

This study has a number of limitations and highlights opportunities for future work.

Although we built a large international cohort of PSP cases with baseline and follow-up imaging, the sample size for vPSP cases was still small (52 PSP–cortical and 17 PSP–subcortical variants). Whilst the SuStaIn algorithm uses a cross-validation framework, ideally, one should have a separate training and test set to validate results. Given the low numbers for the vPSP syndromes in particular, we opted not to do this with our data set so as not to further underpower to model to find patterns associated with these syndromes. Future work will be required to confirm the validity of the two-subtype model on external PSP data sets. We may find that there are further distinct subtypes as vPSP numbers increase, though for this data set, the two-subtype model was optimal. Related to this, we decided to group the PSP–cortical (PSP-F, PSP-S/L, and PSP-CBS) and PSP–subcortical (PSP-P and PSP-PGF) syndromes together in the *post hoc* analyses. This is an established approach in the PSP research setting,^20,33,60,63^ though it will be interesting to look at the finer-grained SuStaIn subtype:clinical syndrome associations when we have larger sample sizes for the vPSP syndromes.

We collected MRI scans from a range of international centres across a number of different scanners. It is well known that using data from different scanners can introduce bias into downstream analyses, through variations in imaging quality, signal homogeneity, and image contrast.^64^ In addition to stringent visual QC of raw images and post-segmentation scans, we also regressed out both scanner manufacturer and field strength when generating adjusted regional volumes to try and account for this. In multi-centre clinical trials of a rare disease such as PSP, the use of multiple scanner types is the rule rather than the exception. We believe that this study’s inclusion of cases across multiple scanner types (albeit adjusted for in the analysis) supports stronger generalizability of the findings to the wider clinical setting.

Another limitation was the use of the Lawton *et al.*^34^ method to convert MoCA scores to MMSE. This method has only been validated in Parkinson’s disease, though it has been used previously in a PSP study.^65^ Given that the MMSE is likely to be less sensitive than the MoCA in PSP,^66^ the cognitive impairment estimated in this study is likely to be on the conservative side.

SuStaIn fits data based on the assumption that there are a distinct set of trajectories, though it is possible that there is a spectrum of disease progression patterns within the data.^1^ In this situation, the identified SuStaIn subtype trajectory could have been created by appending unrelated disease trajectories into one subtype.^26^ When assessing the two-subtype model, we checked the average subtype probability by stage by subtype (Supplementary Fig. 5B), based on the hypothesis that if a separate sequence is appended to another, we would expect the average probability assignment to drop below 50% at some point in the sequence. This was not the case in our model supporting that these are indeed two distinct trajectories. In addition, we found that the majority of individuals remained in the same subtype and progressed to later stages in that subtype which supports the model validity. The high association between PSP–subcortical syndromes and the ‘subcortical’ subtype at baseline could also be due to this caveat, i.e. they don’t necessarily progress through the stages of the subtype to cortical involvement. However, when assessing the stage at follow-up, one of the PSP-SC cases progressed from a baseline stage where there is only subcortical involvement (<stage 13) to a follow-up stage where the cortex started to be involved (>stage 13). Two other cases already had cortical involvement at baseline, progressing to more extensive cortical involvement at follow-up (Supplementary Table 7). Taken together, these findings give confidence that in our model, appending of different disease trajectories into one is unlikely to be the case.

Another consideration is that SuStaIn has no explicit timescale and is only able to extract information on the relative position that the individual is in the sequence within a given subtype. A recently developed generative model called the temporal event-based model addresses this issue, by using longitudinal information to extract transition times between events.^67^ Work to integrate this framework into the SuStaIn algorithm is ongoing. It will be interesting in future work to see whether a temporal SuStaIn model identifies a third subtype, given the finding in this study that the majority of PSP-SC cases follow the same trajectory as PSP-RS cases though with a slower stage progression rate.

## Conclusion

The SuStain model provides data-driven evidence for the existence of two spatiotemporal subtypes of atrophy in clinically diagnosed PSP, giving insights into the relationship between PSP pathology and clinical syndrome. These image-based subtypes are differentially enriched for PSP clinical syndromes and show different clinical characteristics. The results suggest that the PSP-RS and PSP–subcortical syndromes share a similar trajectory of atrophy, though the latter tends to be at an early stage at diagnosis and progresses at a slower rate. Being able to accurately subtype and stage PSP patients at baseline has important implications for screening patients on entry into clinical trials, as well as for tracking disease progression. Future work should focus on validating these results in larger data sets with a higher number of vPSP syndromes that ideally have autopsy-confirmed PSP pathology, extracting information on time to transition between subtype stages, and assessing the clinical relevance of these imaging subtypes in real-world settings.

## Supplementary Material

fcad048_Supplementary_Data

## Data Availability

Source data are not publicly available but non-commercial academic researcher requests may be made to the chief investigators of the seven source studies, subject to data access agreements and conditions that preserve participant anonymity. The underlying SuStaIn model code is publicly available at https://github.com/ucl-pond/pySuStaIn.^68^

## Appendix

### 4RTNI Consortium

**Bradley F. Boeve**—Department of Neurology, Mayo Clinic, Rochester, MN 55905, USA.

**Brad C. Dickerson**—Departments of Neurology and Psychiatry, Frontotemporal Disorders Unit and Alzheimer's Disease Research Center, Boston Massachusetts USA.

**Carmela M. Tartaglia**—Tanz Centre for Research in Neurodegenerative Diseases University of Toronto Toronto Canada.

**Irene Litvan**—Department of Neurosciences, University of California San Diego, La Jolla, California, USA.

**Murray Grossman**—Department of Neurology, University of Pennsylvania, Philadelphia, USA.

**Alex Pantelyat**—Department of Neurology. School of Medicine, Johns Hopkins University, Baltimore, MD, USA.

**Edward D. Huey**—Department of Psychiatry and Neurology, Columbia University, New York, New York, USA.

**David J. Irwin**—Penn Center for Neurodegenerative Disease Research, University of Pennsylvania School of Medicine, Philadelphia, PA, USA.

**Anne Fagan**—Department of Neurology, Washington University School of Medicine, St Louis, MO, USA.

**Suzanne L. Baker**—Molecular Biophysics and Integrated Bioimaging, Lawrence Berkeley National Laboratory, Berkeley, CA, USA.

**Arthur W. Toga**—Laboratory of Neuro Imaging, Stevens Neuroimaging and Informatics Institute, Keck School of Medicine of USC, University of Southern California, Los Angeles, CA, United States.

### PROSPECT Consortium

**Alyssa A. Costantini**, MSc—Department of Clinical and Movement Neurosciences, UCL (University College London) Queen Square Institute of Neurology, London, United Kingdom. a.costantini@ucl.ac.uk.

**Henry Houlden**, FRCP, PhD—Department of Clinical and Movement Neurosciences, UCL (University College London) Queen Square Institute of Neurology, London, United Kingdom; Movement Disorders Centre, UCL Queen Square Institute of Neurology, London, United Kingdom; Department of Neuromuscular Diseases, UCL Queen Square Institute of Neurology, London, United Kingdom. h.houlden@ucl.ac.uk.

**Christopher Kobylecki**, FRCP, PhD—Department of Neurology, Manchester Academic Health Science Centre, Salford Royal NHS (National Health Service) Foundation Trust, University of Manchester, Manchester, United Kingdom. Christopher.Kobylecki@srft.nhs.uk.

**Michele T. M. Hu**, FRCP, PhD—Division of Neurology, Nuffield Department of Clinical Neurosciences, University of Oxford, Oxford, United Kingdom michele.hu@ndcn.ox.ac.uk.

**Nigel Leigh**, FRCP, PhD—Department of Neuroscience, Brighton and Sussex Medical School, Brighton, United Kingdom. P.Leigh@bsms.ac.uk.

## Abbreviations

AD =: Alzheimer’s disease
CBD =: cortico-basal degeneration
CVIC =: cross-validation information criteria
DAV =: davunetide
DC =: diencephalon
DRC =: Dementia Research Centre
EBM =: event-based model
FTLDNI =: frontotemporal lobar degeneration neuroimaging initiative
GIF =: geodesic information flow algorithm
GGT =: globular glial tauopathy
GP =: globus pallidus
LWENC =: Leonard Wolfson Experimental Neurology Centre
MAX =: multiple allocation extinction
MCMC =: Markov Chain Monte Carlo
MDS =: Movement Disorder Society
MMSE =: Mini-Mental State Examination
MOCA =: Montreal Cognitive Assessment
MS =: multiple sclerosis
NA =: nucleus accumbens
NINDS-SPSP =: National Institute of Neurological Disorders and Stroke and Society for Progressive Supranuclear Palsy
PROSPECT =: PROgressive Supranuclear Palsy CorTico-Basal Syndrome Multiple System Atrophy Longitudinal Study
PSP =: progressive supranuclear palsy
PSP-CBS =: PSP–corticobasal syndrome
PSP-F =: PSP–frontal
PSP-P =: PSP–parkinsonism
PSP-PGF =: PSP–primary gait freezing
PSP-RS =: PSP–Richardson
PSP-SL =: PSP–speech/language syndrome
PVD =: positional variance diagram
4R =: four repeat
4RTNI1 =: 4R Tauopathy Imaging Initiative Cycle 1
4RTNI2 =: 4R Tauopathy Imaging Initiative Cycle 2
ROI =: region of interest
SAL =: salsalate
SCP =: superior cerebellar peduncles
SD =: standard deviation
SEADL =: Schwab and England Activities of Daily Living
SPM =: statistical parametric mapping
SuStaIn =: Subtype and Stage Inference
TEBM =: temporal event based model
TIV =: total intracranial volume
UCL =: University College London
UPDRS =: Unified Parkinson Disease Rating Scale
vPSP =: variant PSP
YP =: young plasma
