# Correction to: Uncovering spatiotemporal patterns of atrophy in progressive supranuclear palsy using unsupervised machine learning

**DOI:** 10.1093/braincomms/fcad315

**Published:** 2023-11-27

This is a correction to: William J Scotton, Cameron Shand, Emily Todd, Martina Bocchetta, David M Cash, Lawren VandeVrede, Hilary Heuer, PROSPECT Consortium, 4RTNI Consortium, Alexandra L Young, Neil Oxtoby, Daniel C Alexander, James B Rowe, Huw R Morris, Adam L Boxer, Jonathan D Rohrer, Peter A Wijeratne, Uncovering spatiotemporal patterns of atrophy in progressive supranuclear palsy using unsupervised machine learning, *Brain Communications*, Volume 5, Issue 2, 2023, https://doi.org/10.1093/braincomms/fcad048

In the originally published version of this manuscript, there was an error within Figure 2. The significance bar should be across columns marked PSP-RS and PSP-SC, rather than across those marked PSP-C and PSP-SC. Figure 2 should thus read:

**Figure:**
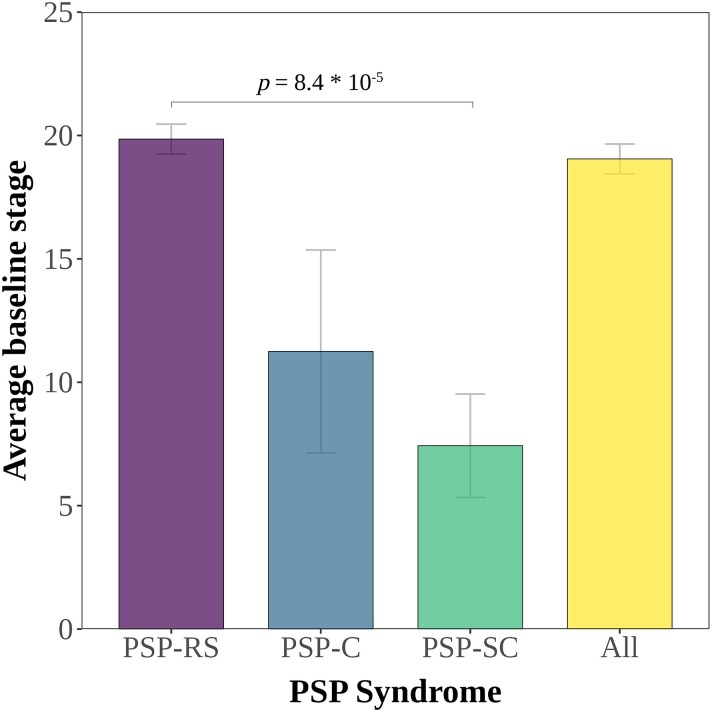


instead of:

**Figure:**
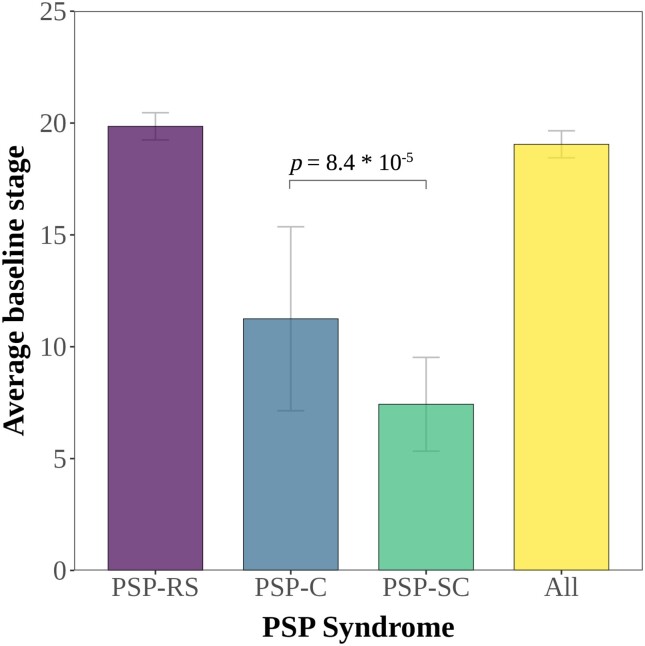


The figure has been emended in the article.

