# Supplementary material for: Uncovering spatiotemporal patterns of atrophy in progressive supranuclear palsy using unsupervised machine learning
Source: Brain Commun. 2023 Mar 2;5(2):fcad048. doi: 10.1093/braincomms/fcad048 (PMC10016410; doi:10.1093/braincomms/fcad048)
Supplement: fcad048_Supplementary_Data [file fcad048_supplementary_data.pdf]

Supplementary Material

Details of cohorts

4RTNI2: Four Repeat Tauopathy Neuroimaging Initiative (Cycle 2)

Participants were recruited from eight sites across North America, as part of a longitudinal observational study of Corticobasal Syndrome (CBS), Progressive Supranuclear Palsy (PSP) or Oligo- or Variant- Progressive Supranuclear Palsy (o/vPSP). The study was managed by the University of California San Francisco (UCSF) with patients also recruited from the University of California of San Diego (UCSD), John Hopkins University, Harvard University Massachusetts General Hospital, the Mayo Clinic Rochester, Columbia University, University of Pennsylvania, and the University of Toronto. A common study design and protocol was run at all sites, and patients were diagnosed with a clinically probable or possible PSP syndrome according to the Movement Disorder Society 2017 PSP diagnostic criteria[1]. All participants had to be aged between 45 – 80yrs to be considered for inclusion, and were evaluated at baseline, 6, 12 and 24 months with a volumetric MRI brain scan, a clinical assessment that included a PSPRS score, UPDRS, and MOCA, Tau and Amyloid PET scans, eye movement function and retinal imaging.

Four scanner types (all 3T) were used in the 4RTNI2 study. A GE Discovery MR750 system (GE, Milwaukee, WI) equipped with 8-channel head coil; whole brain images were acquired with a 3D volumetric inversion recovery fast spoiled gradient-echo recalled sequence (IR-FSPGR; repetition time (TR)/echo time (TE)/inversion time (TI) = 2300/2.98/400 ms, α = 11°, 1.2 mm slice thickness). A GE Signa Premier System (GE, Milwaukee, WI) equipped with 48-channel head coil, whole brain images were acquired with a 3D volumetric inversion recovery fast spoiled gradient-echo recalled sequence (IR-FSPGR; repetition time (TR)/echo time (TE)/inversion time (TI) = 2300/2.98/900 ms, α = 8°, 1.2 mm slice thickness). A Philips Achieva system (Andover, Massachusetts, United States) equipped with 32-chanel head coil, whole brain images were acquired with a volumetric magnetization prepared rapid gradient-echo sequence (MPRAGE; repetition time (TR)/echo time (TE)/inversion time (TI) = 2300/2.98/900 ms, α = 9°, 1 mm slice thickness). A Siemens Magnetom Prism Fit system with a 32-channel receiver head coil was used; whole brain images were acquired with a volumetric

magnetization prepared rapid gradient-echo sequence (MPRAGE; repetition time (TR)/echo time (TE)/inversion time (TI) = 2300/2.91/900 ms, α = 9°, 1.2 mm slice thickness). For each patient, baseline and follow-up MRI were acquired on the same scanner using the same sequence parameters.

For all other cohorts please refer previously published details in Scotton et al. 2022[2].

Supplementary Figures

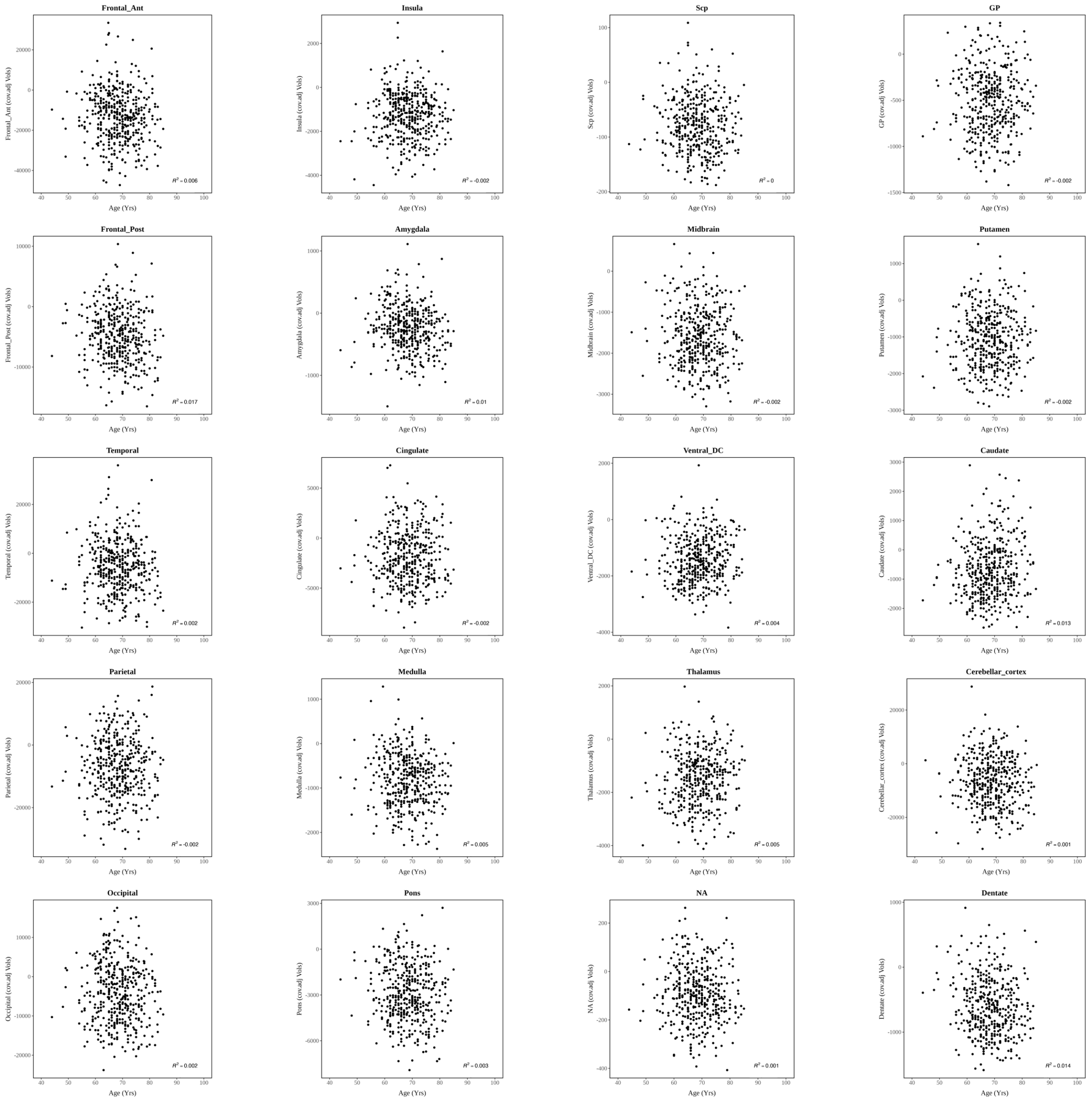

Supplementary Fig. 1: Association of age at baseline scan with covariate adjusted regional volumes in cases. Each scatter plot (20 regions in total) shows every PSP case (n=426) plotted as a function of age at baseline scan (x axis) and covariate adjusted regional volume (y axis). The R^2 represents the proportion of the variation in the covariate adjusted regional volume that is explained by age at baseline scan (linear model = cov. adj. regional vol. ~ age at baseline scan).

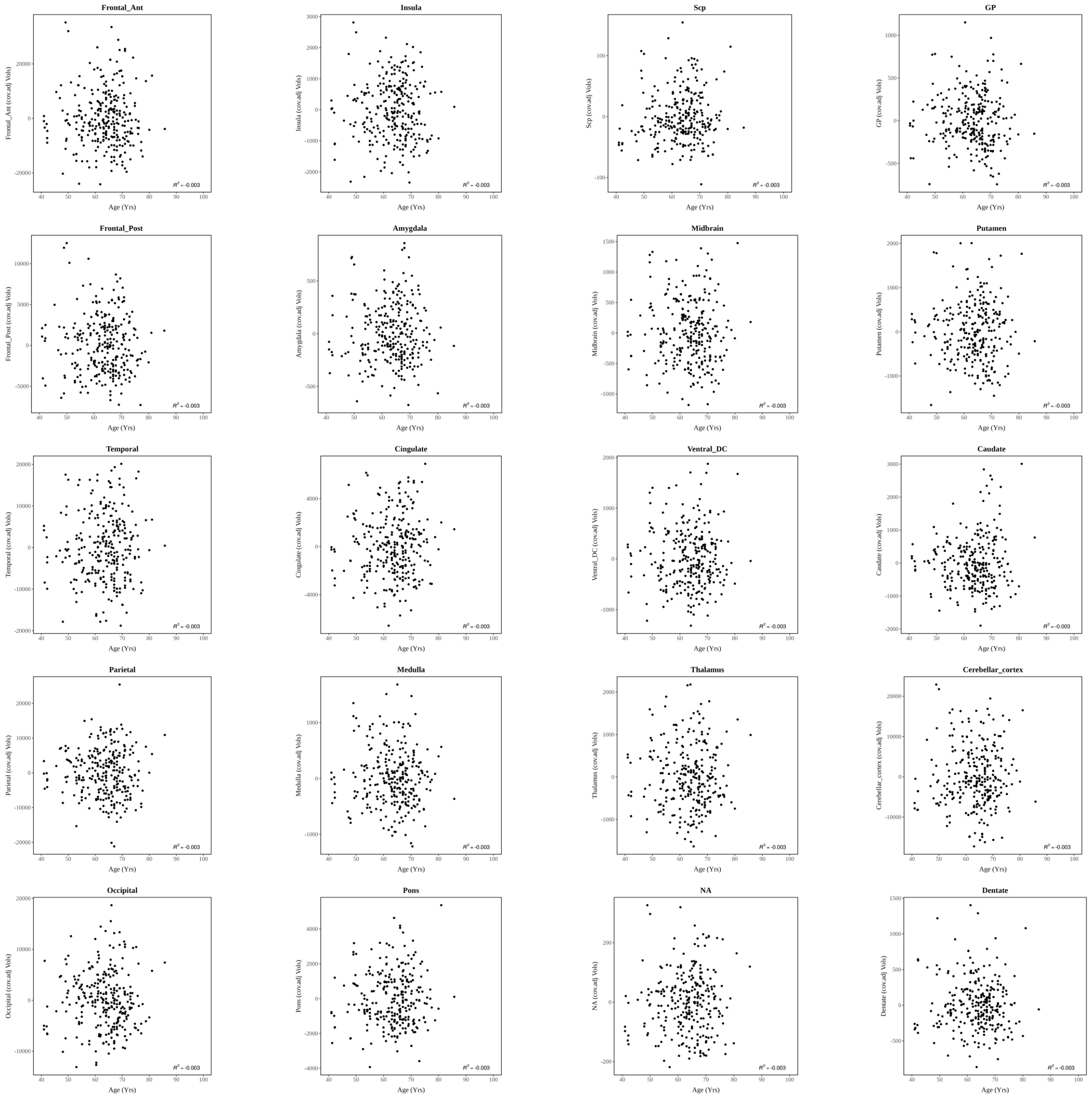

Supplementary Fig. 2: Association of age at baseline scan with covariate adjusted regional volumes in controls. Each scatter plot (20 regions in total) shows every PSP case (n=426) plotted as a function of age at baseline scan (x axis) and covariate adjusted regional volume (y axis). The R^2 represents the proportion of the variation in the covariate adjusted regional volume that is explained by age at baseline scan (linear model = cov. adj. regional vol. ~ age at baseline scan).

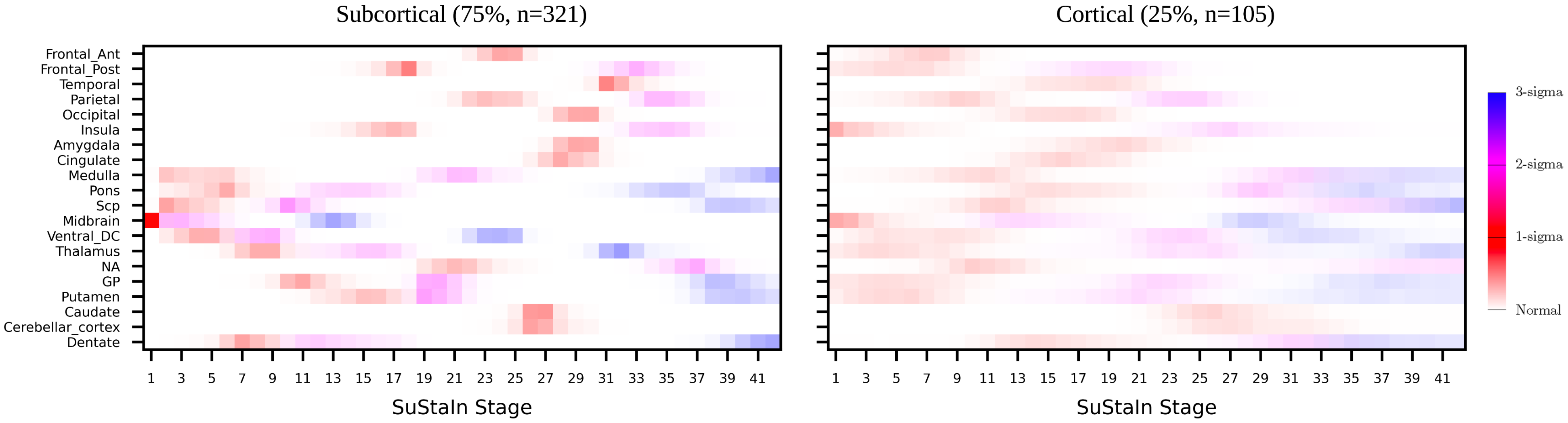

Supplementary Fig. 3: Selecting optimal SuStaIn subtype model given data. Top row shows the cross-validation information criterion (CVIC) plots for **(A)** the 2-subtype model and **(B)** the 3-subtype model. The bottom row shows the log-likelihood across 10 CV folds for each model. The CVIC for the 3-subtype model for the 3-subtype model is very similar to the 2-subtype model and so for parsimony we selected the 2-subtype model as the best description of the data.

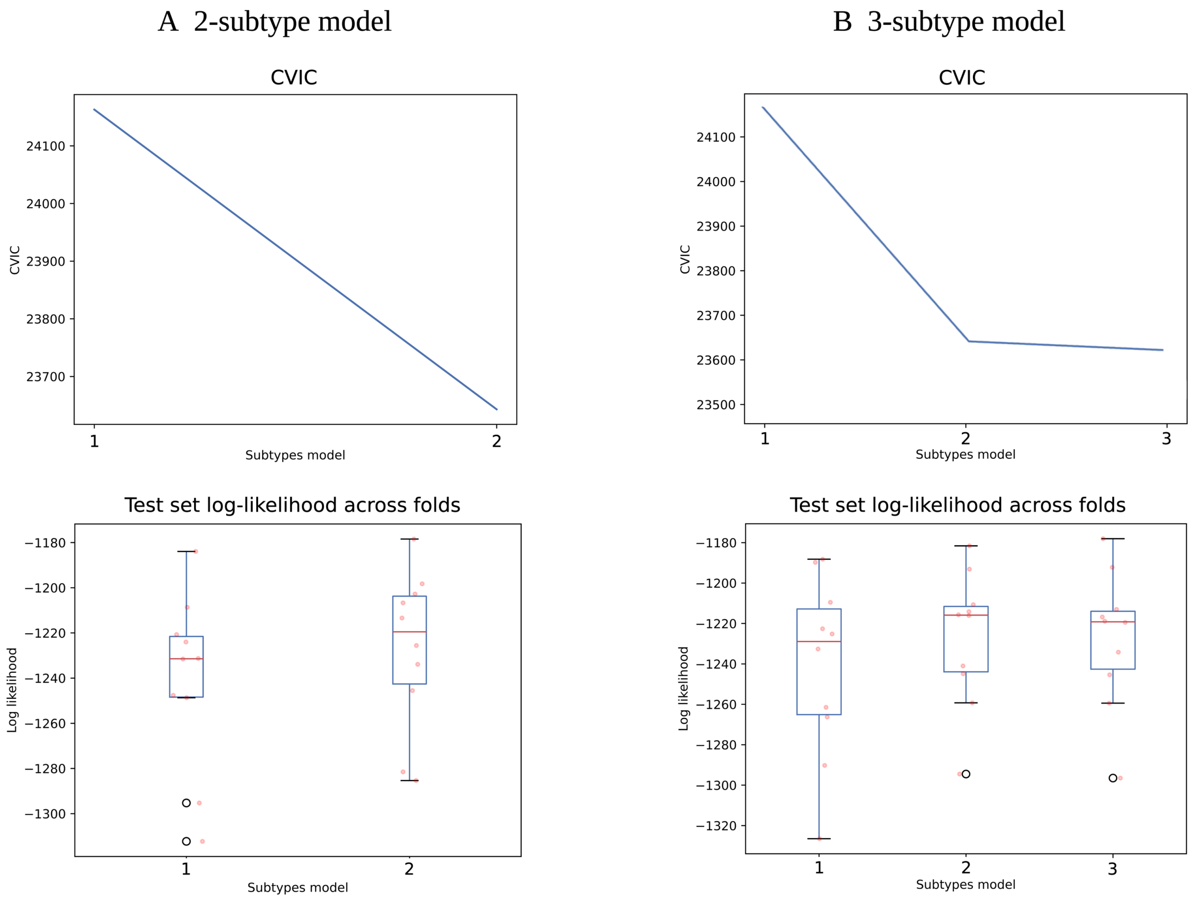

6

Supplementary Fig. 4: Positional variance diagrams for SuStaIn subtypes. These represent the uncertainty in the subtype progression patterns for each region. Each region (y-axis) is shaded based on the probability a particular z score is reached at a particular SuStaIn stage (x-axis). Z scores range from zero (white), one (red), two (pink) to three (blue). Frontal_Ant = anterior frontal lobe, Frontal_Post = posterior frontal lobe (supplementary motor cortex and pre-central gyrus), SCP = superior cerebellar peduncle, NA = nucleus accumbens, GP = globus pallidus.

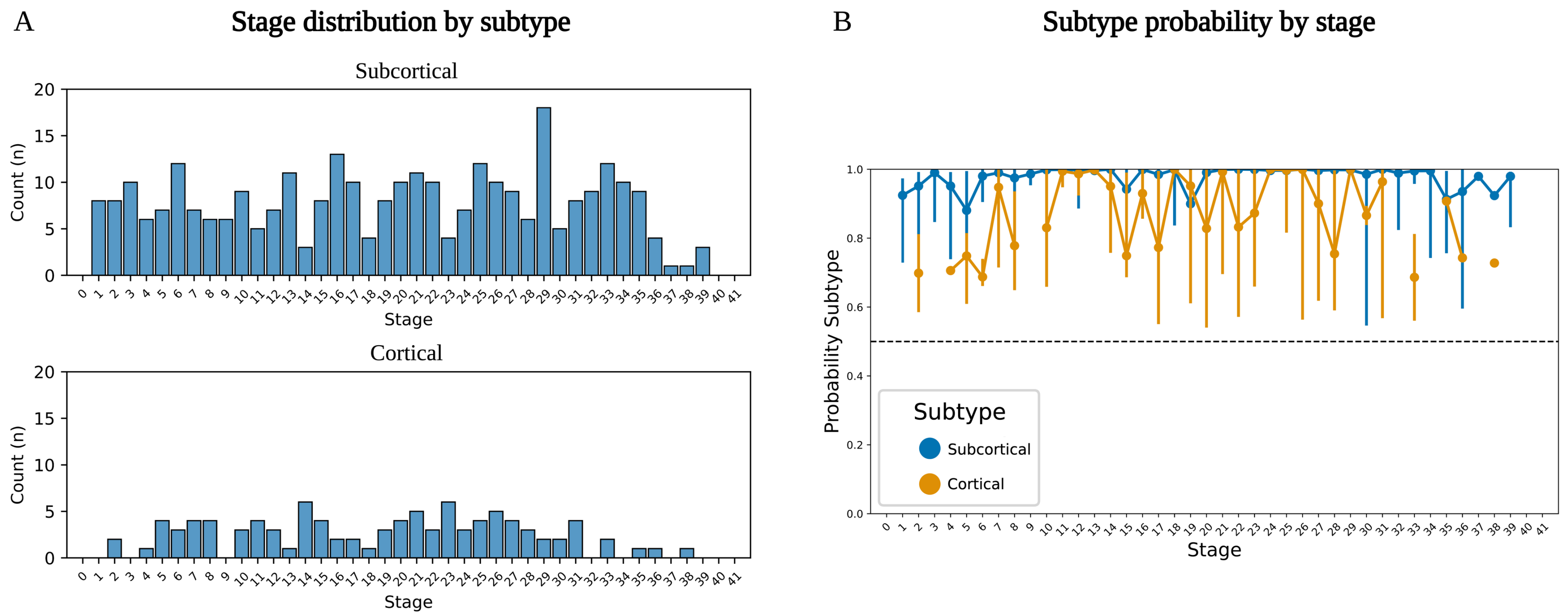

Supplementary Fig. 5: (A) Stage distribution by Subtype for subtypable baseline scans **(B)** Subtype assignment probability by stage for subtypable baseline scans. Dots = median probability, vertical-coloured lines = 95% confidence intervals. The horizontal dotted line represents 50% assignment probability. If any stage had less than 50% probability of assignment to subtype would raise concern that a different underlying disease sequence had been appended to another to make one sequence by SuStaIn.

Supplementary Tables

Supplementary Table 1: Clinical phenotypes and baseline characteristics

	Controls	All	4RTNI1	4RTNI2	DAV	YP/SAL	PROSPECT	UCL
Baseline, n (fu visits)	290	426 (367)	59 (83)	100 (58)	173 (157)	13 (24)	52 (25)	29 (20)
- PSP-RS	-	357 (329)	59 (83)	59 (34)	173 (157)	13 (24)	30 (17)	23 (14)
- PSP-C	-	52 (25)	-	41 (24)	-	-	9 (0)	2 (1)
- PSP-SC	-	17 (13)	-	-	-	-	13 (8)	4 (5)
Sex, % female	56%	48%	54%	48%	51%	48%	42%	31%
Age first scan, y	62.5 (9.4)	68.4 (6.8)	70.2 (7.2)	68.3 (7.0)	67.6 (6.4)	70.0 (3.8)	70.0 (8.1)	67.3 (5.2)
Age first symptom, y[a]	-	64.1 (7.5)	64.7 (7.6)	63.2 (7.5)	-	-	66.3 (8.0)	62.6 (5.1)
Disease duration, y[a, b]	-	4.5 (3.1)	5.4 (3.9)	4.8 (3.3)	-	-	3.8 (2.4)	4.3 (2.6)
Pathology (% PSP)	-	31 (94%)	7 (88%)[c]	-	-	-	6 (100%)	18 (95%)[d]

Values are mean (SD) or n (%), apart from Gender % female, Baseline n (n follow-up visits), Pathology n (% PSP).
4RTNI1 = 4-repeat tauopathy neuroimaging initiative (Phase 1), 4RTN2 = 4-repeat tauopathy neuroimaging initiative (Phase 2), DAV = Davunetide trial, YP = Young plasma trial, SAL = Salsalate trial, PROSPECT = PROgressive Supranuclear Palsy CorTico-Basal Syndrome Multiple System Atrophy Longitudinal Study, UCL = University College London Dementia Research Centre fronto-temporal dementia cohort

[a] note incomplete data for disease duration / age at first symptom.

[b] time from first symptom to first scan.

[c] one case Globular Glial Tauopathy pathology

[d] one case CBD pathology

Supplementary Table 2. List of GIF subregions included in each region used as SuStaIn input.

Regions included in SuStaIn	GIF Subregions
Frontal Anterior	Frontal operculum, central operculum, frontal pole, gyrus rectus, middle frontal cortex, subcallosal area, superior frontal gyrus medial segment, superior frontal gyrus, middle frontal gyrus, opercular part of the inferior frontal gyrus, orbital part of the inferior frontal gyrus, triangular part of the inferior frontal gyrus, anterior orbital gyrus, medial orbital gyrus, lateral orbital gyrus, posterior orbital gyrus
Frontal Posterior	Precentral gyrus, precentral gyrus medial segment, supplementary motor cortex
Temporal	Entorhinal area, fusiform gyrus, parahippocampal gyrus, inferior temporal gyrus, middle temporal gyrus, superior temporal gyrus, temporal pole, planum polare, planum temporale, transverse temporal gyrus
Parietal	Precuenus, parietal operculum, supramarginal gyrus, superior parietal lobule, angular gyrus, postcentral gyrus, postcentral gyrus medial segment
Occipital	Cuneus, calcarine cortex, lingual gyrus, occipital fusiform gyrus, superior occipital gyrus, inferior occipital gyrus, middle occipital gyrus, occipital pole
Insula	Anterior insula, posterior insula
Amygdala	Amygdala
Cingulate	Anterior cingulate gyrus, middle cingulate gyrus, posterior cingulate gyrus
Medulla	Medulla
Pons	Pons
Superior Cerebellar Peduncles	Superior cerebellar peduncles
Midbrain	Midbrain
Ventral Diencephalon	Ventral Diencephalon (GIF segmentation includes subthalamic nucleus, substantia nigra and hypothalamus)
Thalamus	Thalamus
Nucleus Accumbens	Nucleus accumbens
Globus Pallidus	Globus Pallidus
Putamen	Putamen
Caudate	Caudate
Cerebellar Cortex	Lobules I/IV, V, VI, VIIA-Crus I, VIIA-Crus II, VIIB, VIIIA, VIIB, IX, X
Dentate	Dentate nucleus

Supplementary Table 3. Effect size by region of interest.

Region of Interest	Cohen's d
Midbrain	2.56
Ventral diencephalon	1.97
SCP	1.73
Pons	1.72
Thalamus	1.67
GP	1.58
Dentate	1.53
Putamen	1.51
Medulla	1.50
Insula	1.27
Frontal Posterior	1.21
Frontal Anterior	1.10
NA	0.87
Parietal	0.85
Cerebellar cortex	0.79
Caudate	0.78
Amygdala	0.74
Cingulate	0.73
Occipital	0.62
Temporal	0.60
Corpus callosum	0.57[a]
Vermis	0.53[a]
Basal forebrain	0.41[a]
Hippocampus	0.36 [a]

Cohen's d calculated as the standardised mean difference between adjusted the regional volume of that that region of interest (ROI) in cases vs controls.

[a] Cohen's d threshold of greater than or equal to 0.6 was used to select region of interest as input for SuStaIn algorithm resulting in 20 biomarkers being included in model

Supplementary Table 4: SuStaIn algorithm settings for each biomarker

Biomarker	R	Z_{max}
Frontal Anterior	1	4
Frontal Posterior	2	4
Temporal	1	4
Parietal	2	5
Occipital	1	4
Insula	2	4
Amygdala	1	4
Cingulate	1	3
Medulla	3	5
Pons	3	5
SCP	3	5
Midbrain	3	6
Ventral DC	3	6
Thalamus	3	6
NA	2	4
GP	3	5
Putamen	3	4
Caudate	1	4
Cerebellar cortex	1	4
Dentate	3	5

$\boldsymbol{R}$ is the number of z-scores included for biomarker i, and $\mathbf{Z}_{max}$ is the maximum z-score modelled for biomarker i.
Total number of biomarkers (i) for model = 42
$\boldsymbol{C}_{max}$ (the maximum number of subtypes fitted) = 4
Model uncertainty was estimated using 100,000 Markov Chain Monte Carlo (MCMC) iterations.
In the single-cluster expectation maximisation procedure the single-cluster sequence was optimised from 24 different random starting sequences to find the maximum likelihood solution.
Abbreviations: DC = diencephalon, GP = globus pallidus, NA = nucleus accumbens.

Supplementary Table 5: Linear models of covariate adjusted regional volumes by age of scan (Cases)

Region of Interest	Estimate	Standard Error	t value	R^2	p value[a]
Frontal Anterior	-167.1	91.5	-1.83	0.006	1.00
Frontal Posterior	-88.6	30.7	-2.89	0.017	0.08
Temporal	-104.3	74.4	-1.40	0.002	1.00
Parietal	-12.9	66.8	-0.19	-0.002	1.00
Occipital	-69.7	54.6	-1.28	0.002	1.00
Insula	0.7	7.4	0.10	-0.002	1.00
Amygdala	-5.8	2.5	-2.30	0.010	0.44
Cingulate	2.9	18.7	0.16	-0.002	1.00
Medulla	-7.2	4.2	-1.73	0.005	1.00
Pons	-19.9	13.0	-1.53	0.003	1.00
Scp	-0.3	0.4	-0.93	0.000	1.00
Midbrain	-1.7	5.0	-0.33	-0.002	1.00
Ventral DC	9.5	5.8	1.62	0.004	1.00
Thalamus	12.4	7.1	1.74	0.005	1.00
NA	-0.9	0.8	-1.15	0.001	1.00
GP	1.2	2.5	0.47	-0.002	1.00
Putamen	3.1	5.5	0.56	-0.002	1.00
Caudate	17.9	6.9	2.59	0.013	0.20
Cerebellar cortex	-78.5	64.3	-1.22	0.001	1.00
Dentate	-8.1	3.1	-2.60	0.014	0.19

A linear model was fit to each region of interest covariate adjusted volumes (ROI ~ age at scan).
[a] p values were Bonferroni corrected for multiple comparisons
Abbreviations: DC = diencephalon, GP = globus pallidus, NA = nucleus accumbens, ROI = region of interest

Supplementary Table 6: Linear models of covariate adjusted regional volumes by age of scan (Controls)

Region of Interest	Estimate	Standard Error	t value	R^2	p value[a]
Frontal Anterior	-9.6E-10	65.5	-1.5E-11	-0.003	1
Frontal Posterior	2.5E-09	22.0	1.1E-10	-0.003	1
Temporal	-6.5E-09	50.3	-1.3E-10	-0.003	1
Parietal	-1.8E-09	42.9	-4.1E-11	-0.003	1
Occipital	1.3E-08	35.2	3.6E-10	-0.003	1
Insula	1.6E-09	6.0	2.7E-10	-0.003	1
Amygdala	2.0E-10	1.7	1.2E-10	-0.003	1
Cingulate	1.6E-09	16.0	9.7E-11	-0.003	1
Medulla	3.1E-10	3.1	1.0E-10	-0.003	1
Pons	-1.1E-09	9.6	-1.2E-10	-0.003	1
SCP	6.2E-11	0.3	2.4E-10	-0.003	1
Midbrain	2.3E-10	3.2	7.4E-11	-0.003	1
Ventral DC	-3.4E-10	3.5	-9.7E-11	-0.003	1
Thalamus	9.0E-10	4.6	2.0E-10	-0.003	1
NA	7.3E-11	0.6	1.1E-10	-0.003	1
GP	7.7E-11	1.9	4.1E-11	-0.003	1
Putamen	-6.8E-12	4.2	-1.6E-12	-0.003	1
Caudate	9.1E-10	4.8	1.9E-10	-0.003	1
Cerebellar cortex	-6.3E-09	46.4	-1.4E-10	-0.003	1
Dentate	-2.5E-10	2.2	-1.1E-10	-0.003	1

A linear model was fit to each region of interest covariate adjusted volumes (ROI ~ age at scan).
[a] p values were Bonferroni corrected for multiple comparisons
Abbreviations: DC = diencephalon, GP = globus pallidus, NA = nucleus accumbens, ROI = region of interest, SCP = superior cerebellar peduncles

Supplementary Table 7: Average stage by clinical syndrome by SuStaIn Subtype

	Subcortical subtype	Cortical subtype	*p* value[a]
All	19.0 (10.5)	18.3 (9.1)	0.85
PSP-RS	19.9 (10.2)	18.9 (8.7)[b]	0.98
PSP-SC	**7.4 (5.8)**[a, b, c]	12.0 (11.3)	0.98
PSP-C	11.3 (8.9)	17.9 (9.6)	0.54

Mean stage (SD) at baseline
One way ANOVA (Mean stage ~ PSP syndrome + Sustain baseline subtype). PSP syndrome $p = 8.1 \times 10^{-6}$, Sustain baseline subtype $p = 0.98$.
[a] Tukey HSD was used for post-hoc multiple comparisons
[b] Statistically significant at $p < 0.05$, corrected for multiple comparisons.
[c] one case PSP-Subcortical assigned to the *Subcortical* subtype case progressed from stage 12 (confined to subcortical regions to 14 at follow-up (insula and posterior frontal lobe abnormal). Two PSP-Subcortical cases assigned to the *Subcortical* subtype had more extensive cortical involvement at baseline (stage 16 and stage 26 respectively at baseline, and stage 16 and stage 27 at follow-up).
Abbreviations: PSP-RS = PSP Richardson syndrome, PSP-SC = PSP subcortical (includes PSP-parkinsonism and PSP-progressive gait freezing), PSP-C = PSP-cortical (includes PSP-frontal, PSP-speech/language disorder and PSP-corticobasal syndrome)

Bibliography

1. Höglinger GU, Respondek G, Stamelou M, et al. Clinical diagnosis of progressive supranuclear palsy: The movement disorder society criteria. *Mov Disord*. 2017;32(6):853-864. doi:10.1002/mds.26987

2. Scotton WJ, Bocchetta M, Todd E, et al. A data-driven model of brain volume changes in progressive supranuclear palsy. *Brain Commun*. 2022;4(3). doi:10.1093/braincomms/fcac098
